# How Intense Is Effective? Exploring Aerobic Exercise Intensity for Knee Osteoarthritis Through a Bayesian NetworkMeta-Analysis

**DOI:** 10.3390/healthcare14040451

**Published:** 2026-02-11

**Authors:** Luis Cabrera-Durán, Javier Palomares-Fernández, Ignacio Canitrot-González, Paride Crisafulli, Carlos Donato Cabrera-López, José Fierro-Marrero

**Affiliations:** 1 Departamento de Fisioterapia, Centro Superior de Estudios Universitarios La Salle, Universidad Autónoma de Madrid, 28023 Madrid, Spain; luiscd.fisio@gmail.com (L.C.-D.); jvrpalomares1@gmail.com (J.P.-F.); nachocani10@gmail.com (I.C.-G.); jose.fierromarrero@yahoo.com (J.F.-M.); 2 IFISC, Instituto de Física Interdisciplinar y Sistemas Complejos (CSIC-UIB), Campus Universitat de les Illes Balears, 07122 Palma de Mallorca, Spain; paride@ifisc.uib-csic.es; 3 Clínica de Fisioterapia FisioKnee, Calle Ros de Olano 5, 28002 Madrid, Spain; 4 Motion in Brains Research Group, Centro Superior de Estudios Universitarios La Salle, Universidad Autónoma de Madrid, 28049 Madrid, Spain

**Keywords:** knee osteoarthritis, knee pain, aerobic exercise, exercise intensity, network meta-analysis

## Abstract

Introduction: Knee osteoarthritis (KOA) is characterized by pain, stiffness, and functional limitation. Aerobic exercise (AE) is a key treatment with proven benefits. Aims: To determine the most effective AE intensity for KOA. Methods: Searches were performed in seven databases, including randomized controlled trials with AE-only groups. Outcomes assessed were pain, walking and sit-to-stand performance, stiffness, and disability. Methodological quality and bias were evaluated. Bayesian random-effects network meta-analyses compared AE intensities, reporting standardized mean differences (Hedges’ g) with 95% credible intervals. Certainty of evidence was rated using GRADE. Results: Fifteen studies were included (mean PEDro score: 5.93), showing “some concerns” or “high risk” of bias. Two meta-analyses (pain and walking performance) were conducted. Comparisons between AE intensities showed non-significant, imprecise results with very low certainty of evidence. Conclusions: Evidence is insufficient to identify the optimal AE intensity for KOA. Limitations include high risk of bias, wide credible intervals, and reliance on indirect comparisons. In this context, clinicians should apply a structured, patient-centered approach to AE prescription, considering gradual progression and monitoring of tolerance, and combine AE with other recommended interventions. High-quality trials with direct comparisons of AE intensity should be conducted for this inconclusive gap.

## 1. Introduction

According to the Osteoarthritis Research Society International, osteoarthritis (OA) is a disease of articular cartilage characterized by progressive degradation. OA first manifests as a molecular disorder (abnormal joint tissue metabolism) and later as an anatomical and/or physiological disorders, such as cartilage degradation, bone remodeling, osteophyte formation, joint inflammation, and loss of normal joint function [1]. Symptoms vary in intensity but usually worsen over time. The most common are pain, stiffness, and swelling. Disease severity can be classified radiologically using the Kellgren–Lawrence system [2].

Knee osteoarthritis (KOA) has a global prevalence of 22.9% of individuals over 40 years, diagnosed through radiological or symptomatic–radiological criteria [3]. The global incidence is 203 per 10,000 person-years among individuals aged 20 and above, higher for radiological than symptomatic diagnosis. Both prevalence and incidence increase with age and are higher in women [3]. KOA generates relevant healthcare costs, reaching an average annual cost of 2295 € per patient, rising with joint degeneration [4]. In the United States, costs can reach 10,000 € per patient, a difference potentially attributed to the higher obesity prevalence [5].

Current clinical practice guidelines recommend a multimodal approach in KOA, combining exercise, education, and weight control [6]. Notably, all six of the most recent clinical practice guidelines strongly recommend the use of exercise therapy regardless of age, pain, or osteoarthritis severity, with several guidelines recommending exercise as a first-line intervention [6]. Exercise therapy is considered safe, as different exercise modalities are not associated with an increased risk of adverse events [7].

Despite this strong recommendation, current guidelines do not provide information on how exercise therapy should be prescribed, particularly with respect to modality selection and dosage parameters [6]. Although the clinical benefits of exercise for KOA are well supported, key questions regarding optimal prescription remain unresolved [6].

Exercise is a cost-effective intervention, yet uncertainty persists regarding the most effective exercise modality and its optimal dosage. Recent research has examined a range of exercise modalities, including aerobic, resistance, mind–body, flexibility-based programs, and their combinations, through several time-points [7]. A generalized finding observed in this meta-analysis concerns that exercise modalities seem to modify pain intensity and functioning. However, results are imprecise for its effectiveness in quality of life, remaining uncertain. When checking for the specific effects of modalities, the majority seem to provide consistent effects on pain and functioning when interventions are provided for approximately 12 weeks, with uncertainty for its effectiveness in short and long interventions (aprox. 4 and 24 weeks respectively). However, AE seems to be a promising modality as we currently consider evidence to support its effectiveness with short-term interventions, being the only one to show consistent findings at this time point [7].

Among these modalities, AE has been highlighted as particularly promising due to its additional cardiovascular and anti-inflammatory benefits, which may be especially relevant in populations with KOA and frequent comorbidities [8]. Accordingly, several studies have begun to explore exercise dosage parameters using frameworks such as the FITT-VP model, examining factors such as exercise intensity, session duration, weekly frequency, intervention length, and supervision. For example, Juhl et al. [9] reported that supervised exercise was associated with improved outcomes in certain modalities, including AE, whereas no specific dosage parameters significantly influenced the effects of resistance exercise in populations with KOA, with and without hip OA.

Nevertheless, evidence regarding the role of exercise intensity remains inconsistent. A recent meta-analysis reported no clinically significant differences according to exercise intensity in either aerobic or resistance modalities [10], though findings were characterized by substantial heterogeneity and imprecision.

Importantly, no meta-analysis to date has specifically evaluated the clinical effectiveness of isolated AE across different intensity levels in isolated KOA populations. Given the widespread recommendation, safety profile, feasibility, and scalability of AE, clarifying whether exercise intensity influences its effectiveness represents a relevant and unresolved research question. The present study proposes to fill of this gap by conducting a network meta-analysis, where different categories of AE intensities will be compared directly and indirectly through a network meta-analysis.

To address this gap, the aim of the present study is to compare the effects of different AE intensities on clinical and functional outcomes exclusively in KOA populations. We conducted a Bayesian network meta-analysis, which allows the integration of both direct and indirect comparisons between multiple intensity categories.

## 2. Methods

This systematic review and network meta-analysis (NMA) followed the Preferred Reporting Items for Overviews of Systematic Reviews and Meta-analysis Network Meta-analysis Extension Statement [11]. The protocol was also registered in PROSPERO (CRD42024519632 on 12 March 2024).

### 2.1. Selection Criteria

Selection criteria were based on the PICOS strategy.

#### 2.1.1. Population

Patients with KOA were eligible regardless of the diagnostic criteria. Studies combining KOA and other concomitant musculoskeletal pathologies, such as hip OA, were excluded.

#### 2.1.2. Intervention

Eligible interventions should solely consist of AE alone. Studies combining exercise with any other intervention such as lifestyle recommendation or educational protocols were excluded. Studies should include internal load parameters of AE intensity, such as heart rate (HR), oxygen consumption measures, or subjective internal intensity parameters like perceived exertion or talk test.

#### 2.1.3. Comparison

Any type of comparator was eligible for inclusion. Comparison groups would serve as connectors through the network.

#### 2.1.4. Outcome Measures and Time-Point Analysis

The following outcome measures were considered for inclusion: (1) knee pain intensity; (2) performance in walking tasks; (3) performance in sit-to-stand tasks; (4) performance in combined sit-to-stand and walking tasks (timed-up-and-go or similar); (5) perceived knee stiffness; (6) disability related to KOA, exclusively analyzed with the total points in the Western Ontario McMaster Universities Osteoarthritis Index (WOMAC). Only immediate post-intervention analyses were eligible.

#### 2.1.5. Data Availability and Study Design

Eligible studies should present sample size, central tendency, and dispersion parameters for the outcomes and time-point of interest. Only randomized controlled trials were eligible.

### 2.2. Search Strategy

Systematic searches were conducted using PubMed, EBSCO, Web of Science, SciELO, ScienceDirect, Scopus, and Google Scholar to find eligible studies across multiple databases. Search engines, search equations, databases, number of registries retrieved, and search dates are reported in the Supplementary Material. Additionally, non-systematic manual and citation searches were performed.

### 2.3. Selection Process

The selection process was conducted by two researchers (LCD and ICG) using Rayyan.ai [12]. Duplicates were identified automatically and manually removed. Reviewers independently and blindly screened records in two stages. For the first stage, Title–Abstract–Keywords–Design were analyzed. A pilot test with the first 60 records refined criteria: the first 30 were analyzed, discussed with the group, reanalyzed, and then reapplied the discussed criteria to records 31–60, which were additionally reanalyzed. The remaining records were screened, with final disagreements resolved by discussion of both reviewers or with a third reviewer when needed. In the second stage, records selected for full text analysis were reassessed. Final disagreements were resolved by discussion of both reviewers or with a third reviewer when needed.

Additional searching methods, such as manual and citation searches, were conducted collaboratively by the reviewers.

### 2.4. Data Extraction

#### 2.4.1. Summary Information

The summary information would include authors, study design, KOA diagnostic criteria and the presence of pain. Additionally, the BMI, diabetes, and blood pressure values would be provided. Experimental (aerobic) and control groups (other interventions), allocated and analyzed sample size, age, and sex within groups would be provided.

Only the previous six outcome measures of interest would be extracted, along with their assessment tools. The results of the comparison between experimental groups or experimental with control groups for post-intervention analysis would be extracted. Summary results of this information were presented narratively and schematically indicating the direction of the effect (>, <, or ≈between groups).

#### 2.4.2. Exercise Prescription

AE prescription parameters were extracted. These include the exercise activity (walking, cycling, etc.), and density features (intervallic or continuous modalities). In addition, volume parameters would be reported, including:

- Total session duration (including exercise and rest periods).
- Stimuli duration (specific time spent exercising).
- Weekly frequency.
- Number of weeks.

Additionally, the intensity of AE, monitorization instruments, and the intensity progression parameters would be reported. Exercise intensity parameters would be categorized into “near-maximal”, “vigorous”, “moderate”, “light”, “very light”, or any of their combinations (such as very-light-to-light, etc.) following the criteria by the American College of Sports Medicine [13] for percentage of maximum heart rate (%HRmax), percentage of maximum heart rate reserve (%HRR), percentage of maximal oxygen consumption (%VO_2_max), percentage of oxygen consumption reserve (%VO_2_reserve), and Borg 6–20 scale, see Supplementary Material. Estimations for other instruments such as the Borg CR-10 would be conducted based on the conversions proposed by Borg [14]. Talk test zones would be identified [15] to estimate HR or oxygen consumption measures, and consequently the intensity [16].

#### 2.4.3. Data Extraction Procedure

Three researchers independently and blindly extracted the following information: (1) type of intervention of any group; (2) AE intensity prescription; (3) categorization of exercise intensity into “near-maximal”, “vigorous”, “moderate”, “light”, “very light”, or their combinations; (4) outcome measures of interest, and the instruments/tests employed; (5) sample size analyzed, central tendency and dispersion results. The agreed upon information was considered valid, included in the summary table, and used for meta-analyses. Disagreements would be resolved between reviewers. The remaining information was extracted collaboratively.

### 2.5. Methodological Quality and Risk of Bias

Two reviewers would blindly and independently assess the methodological quality using the PEDro scale, and the risk of bias using the Risk of Bias Tool 2.0 [17,18]. Agreed on items would be maintained, while disagreements were resolved by the group’s decision.

Inter-rater agreement was tested for each item within every study employing a quadratic weighted Cohen’s kappa coefficient (κ). The interpretation was “almost perfect” for 0.81–1.00; “substantial” if 0.61–0.80; “moderate” if 0.41–0.6; “fair” if 0.21–0.4; “slight” if 0.00–0.20; and “poor” when < 0.00 [19]. The function “cohen.kappa” was employed from the “psych” package version 2.3.12 [20] in R Software version 4.3.1 [21].

### 2.6. Network Meta-Analysis

A network meta-analysis (NMA) was conducted to compare the efficacy of AE across different intensity levels, with “no treatment” serving as the primary control node. Experimental nodes represented specific aerobic exercise intensities classified as near-maximal, vigorous, moderate, light, very light, or any combination of these, following previously established methodology.

Standardized mean differences were calculated using Hedges’ g [22] and interpreted as follows: very small (<0.20), small (0.20–0.49), medium (0.50–0.79), and large (≥0.80) [23]. Two Bayesian NMA models were implemented:

- Model 1: A Bayesian model including only the intervention effect (no covariates).
- Model 2: An adjusted Bayesian model including the intervention effect plus two study-level covariates: intervention duration (number of weeks) and weekly frequency.

The Bayesian hierarchical NMA was implemented using a custom Python script (version 3, see Supplementary Material) whose main class is based on the PyMC3 module [24].

The Bayesian models would produce three main outputs:

1. Network graph indicating the direct connections established within each NMA.
2. Forest plot comparing exercise interventions against the no treatment control.
3. Pairwise comparison between interventions, reported as network (mixed), direct, and indirect estimates. Inconsistency between direct and indirect evidence was tested with a two-tailed z-test.
4. Funnel plots to assess potential publication bias.

A detailed view of the meta-analysis methodology, Hedges’ g calculation, Bayesian models, and the plot generation processes can be found in the Supplementary Material.

### 2.7. Synthesis of Results

The synthesis of results will be presented with the adapted Cochrane GRADE (Grading of Recommendations Assessment, Development and Evaluation) of evidence for NMA [25]. This framework allows drawing conclusions on the certainty of the available evidence provided by the NMA, based on a stepwise evaluation of direct, indirect, and mixed evidence. The certainty is first analyzed respectively for direct and indirect evidence (most dominant loop), with both being tested for risk of bias, heterogeneity, indirectness and publication bias. Additionally, indirect evidence is tested for intransitivity. Intransitivity was assessed qualitatively following GRADE indications, checking for the presence of potential modifiers across direct comparison and indirect loops, such as different populations, interventions, or outcome measures. The final analysis from mixed evidence starts considering the certainty of the most dominant contribution of either direct or indirect evidence. Then, it is assessed for incoherence (z-test between direct and indirect estimates) and imprecision.

Each domain is judged as “not serious”, “serious”, or “very serious”, leading to downgrading the certainty by 0, 1 or 2 levels respectively. Limitations across domains result in an overall rating of the certainty of evidence as high, moderate, low or very low.

## 3. Results

### 3.1. Selection Process

**Figure 1:**
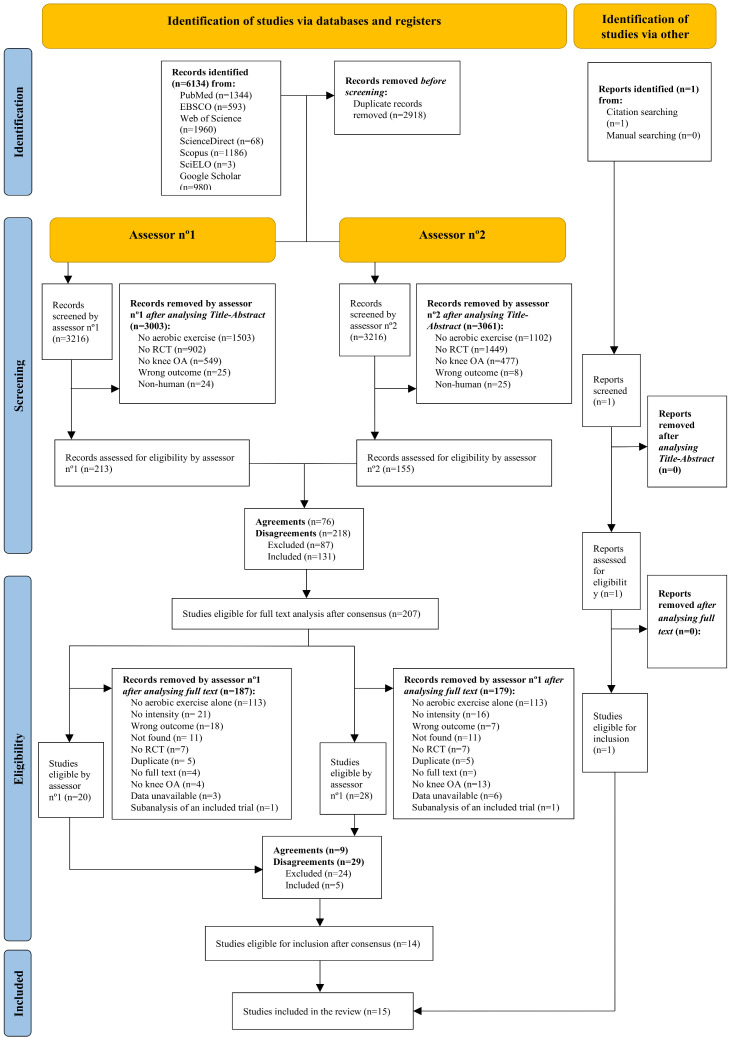
Flowchart of search and selection process.

A total of 6134 records were retrieved from the databases. After removing duplicates, 3216 records were screened for Title–Abstract–Keywords–Design. After resolving discrepancies, 207 records were analyzed in full text. A total of 15 studies [26,27,28,29,30,31,32,33,34,35,36,37,38,39,40] were included in the review, see Figure 1.

### 3.2. Summary Information

Diagnostic criteria varied across studies, with 12 studies selecting patients through both clinical and radiological criteria [26,27,28,30,31,32,35,36,37,38,39,40], only clinical [29], only radiological [34], or with an unstandardized procedure (diagnosed by a surgeon) [33].

The presence of pain was explicitly confirmed in nine studies [27,28,30,31,32,35,36,37,38]. In five other studies, the presence of pain was likely but not clearly stated (inferred from baseline pain assessments) [26,29,34,39,40]. One study did not provide clear information for the presence of pain [33].

A total of 455 patients were assigned to AE groups. Among them, 30 patients participated in a very-light-to-light intensity AE [40]; 20 were assigned to light-to-moderate intensity exercise [35]; 22 were assigned to light-to-moderate-to-vigorous exercise [30,31]; 31 participated in moderate intensity exercise [33,38]; 235 were assigned to a moderate-to-vigorous intensity exercise [27,32,34,36,37]; and 117 engaged in vigorous intensity exercise [26,28,29,33,35,39].

A total of 576 subjects were assigned to control groups. Among these, 259 subjects were allocated to resistance exercise programs [28,30,31,32,36,37,39]; 171 to education-based interventions [30,31,32,36]; 25 to a combined exercise program [34]; 24 to resistance exercise combined with behavioral and lifestyle modification strategies [34]; and 97 to no-treatment groups [27,37,38,39].

AE intensity was monitored using several procedures such as %HRmax [26,27,30,31,34,37,38,40], %HRR [32,35,36,39], the talk test [33], the Borg Rating of Perceived Exertion (6–20 points) [28,30,31,40], or the Borg CR10 scale [29].

The mean number of weeks for AE protocols was 16.9 ± 21.5 (range of 6–79), with a median of 10 (Q1 = 8, Q3 = 13), and a mode of 8 weeks. The weekly frequency of AE sessions was 2.93 ± 0.52 (2–4 range) with a median and mode of 3 sessions per week (Q1 = 3, Q3 = 3).

Twelve studies measured pain intensity [26,28,29,30,31,32,34,36,37,38,39,40], 10 explored walking performance [26,27,29,30,32,33,35,38,39,40], four analyzed sit-to-stand performance [30,33,35,39], three studies analyzed combined walking and sit-to-stand performance [27,33,40], and only five explored perceived knee stiffness [26,29,30,38,39] and disability associated with KOA [29,30,33,38,39]. See Supplementary Material for tabular information of each study included.

### 3.3. Methodological Quality and Risk of Bias

Of the 15 evaluated studies, the average score on the PEDro scale was 5.93 points (ranging from 4 to 8), with moderate-to-perfect reliability. Regarding the ROB 2.0 scale, for pain intensity, eight articles were rated as having “high risk” of bias [26,28,32,34,36,38,39,40], and three presented “some concerns” [29,30,37], extracted with slight-to-perfect reliability.

For walking performance, 10 studies presented “high risk” of bias [26,27,31,32,33,35,36,38,39,40], while one study presented “some concerns” [29], with slight-to-perfect reliability.

All studies evaluating sit-to-stand performance presented a “high risk” of bias [31,33,35,39], determined with slight-to-substantial reliability.

Similarly, all studies assessing combined sit-to-stand and walking performance were rated with “high risk” of bias [27,33,40], with a reliability ranging from fair to moderate.

For the perceived stiffness, three studies showed “high risk” of bias [26,31,38], while one presented “some concerns” [29], with a reliability ranging from fair to substantial.

Finally, regarding disability related to KOA, four studies were rated with a “high risk” of bias [31,33,34,38], while one study presented “some concerns” [29]. Inter-rater agreement ranged from fair to substantial. See Supplementary Material for detailed analysis of PEDro and ROB 2.0.

### 3.4. Network Meta-Analysis

Two meta-analyses were conducted: one focused on pain intensity, and the other on walking performance. Although six outcomes were initially considered, the remaining four could not be analyzed due to insufficient connectivity between studies in the network. Details on the eligibility process for conducting the meta-analyses, data availability, instrument selection, and the estimations into means and SD are presented in the Supplementary Material.

#### 3.4.1. Pain Intensity

**Figure 2:**
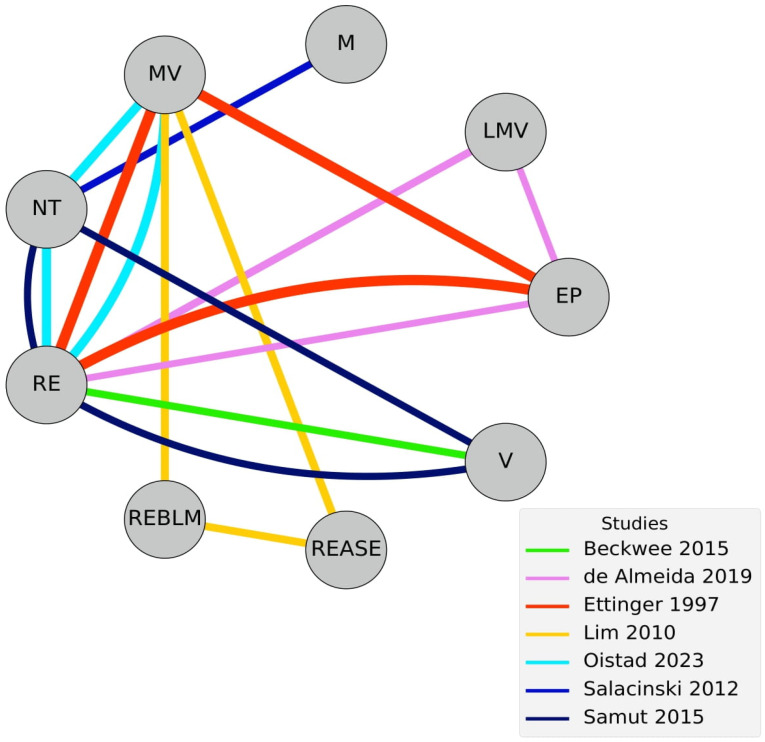
NMA pain netgraph. Connections between nodes present are presented with distinct colors according to the studies conducting such comparisons [28,30,32,34,37,38,39].

Seven studies were included in the meta-analysis [28,30,32,34,37,38,39]. Studies explored light-to-moderate-to-vigorous [30], moderate [38], moderate-to-vigorous [32,34,37] and vigorous intensities [28,39]. No direct comparisons between AE intensities were available. Control groups included educational protocols [30,32], resistance exercise [28,30,32,37,39], no treatment [38], and multimodal exercise (resistance, aerobic and stretching) interventions [34]; see Figure 2.

**Figure 3:**
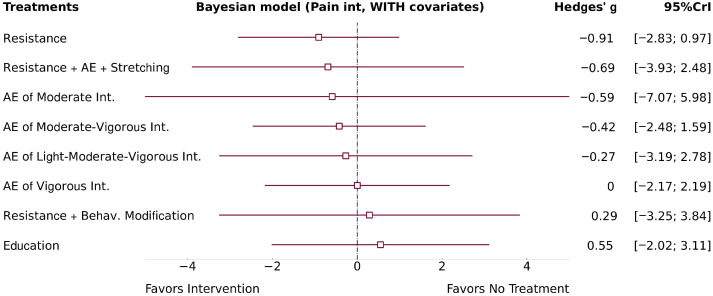
NMA pain forest Model 2.

The two NMA models revealed non-significant and imprecise effects for all AE intensities, including light-to-moderate-to-vigorous (Model 1: g = −0.27; 95% CrI −3.19, 2.67; Model 2: g = −0.27; 95% CrI −3.19, 2.78), moderate (Model 1: g = −0.65; 95% CrI −4.09, 2.99; Model 2: g = −0.59; 95% CrI −7.07, 5.98), moderate-to-vigorous intensities (Model 1: g = −0.42; 95% CrI −2.49, 1.56; Model 2: g = −0.42; 95% CrI −2.48, 1.59), and vigorous (Model 1: g = 0; 95% CrI −2.19, 2.17; Model 2: g = 0; 95% CrI −2.17, 2.19) compared to “no treatment”. Heterogeneity was large in both models—Model 1 (τ = 1.40) and Model 2 (τ = 1.40). Model 1 forest plot is presented in the Supplementary Material; see Model 2 forest plot in Figure 3.

The addition of both covariates (weekly frequency and number of weeks) did not meaningfully adjust the precision of AE intensity estimates. In addition, neither weekly frequency (w_f_ = −0.15; 95% CrI −19.15, 18.28) nor number of treatment weeks (w_n_ = −0.09; 95% CrI −19.34, 19.30) showed a linear association with the observed effects.

Comparisons between different intensities of AE were based exclusively on indirect evidence. All estimated effects from both models were non-significant, with 95% CrI exceedingly wide in both directions, including large effect sizes (g > 0.7). This indicates a high degree of imprecision, limiting reliable conclusions about the true effect of AE intensities; see Table 1.

The preliminary GRADE assessment of the indirect network for both models identified “very serious” concerns regarding risk of bias, but “not serious” for indirectness. Heterogeneity was rated as “very serious”, whereas intransitivity was judged as “not serious”. Publication bias across the indirect network ranged from “not serious” to “very serious” depending on the comparison, see Supplementary Material with funnel plots. For the mixed estimates, imprecision was judged as “very serious” across all comparisons. Consequently, the final GRADE evaluation indicated an overall certainty of evidence of “very low” for all comparisons of AE intensities in both models, see Table 1.

#### 3.4.2. Walking Performance

Seven studies were included in the meta-analysis [27,31,32,33,35,38,39]. Studies explored AE interventions of light-to-moderate [35], light-to-moderate-to-vigorous [31], moderate [33,38], moderate-to-vigorous [27,32], and vigorous intensities [33,35,39]. Two direct comparisons between intensities were available: light-to-moderate vs. vigorous [35]; and moderate vs. vigorous [33].

**Figure 4:**
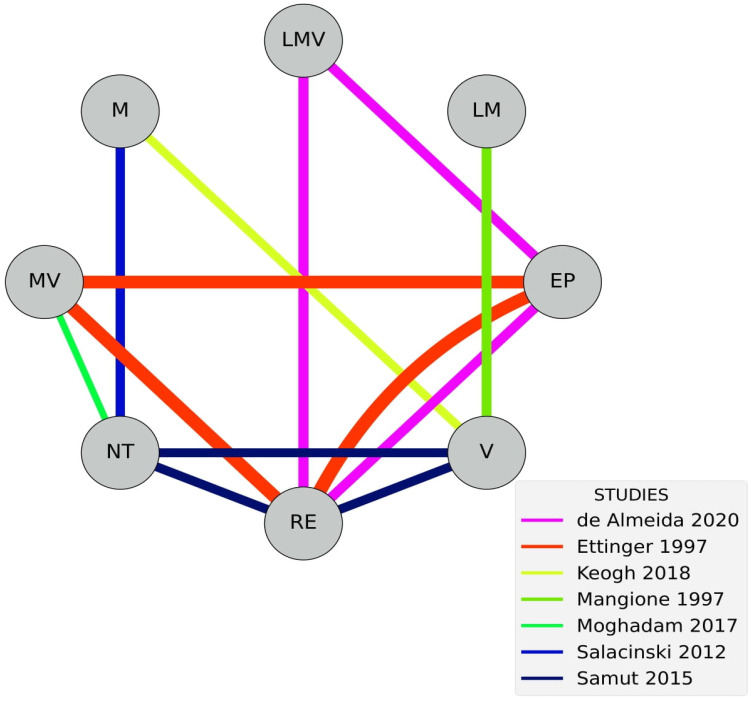
NMA walk netgraph. Connections between nodes present are presented with distinct colors according to the studies conducting such comparisons [27,31,32,33,35,38,39].

Control groups included resistance exercise [28,31,32,34,37,39], educational protocols [31,32,39], and no treatment [27,38]; see Figure 4.

**Figure 5:**
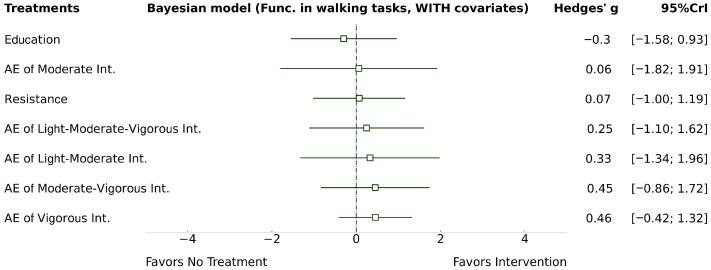
NMA walk forest Model 2.

The two NMA models revealed non-significant and imprecise effects for all AE intensities, including light-to-moderate intensity (Model 1: g = 0.78; 95% CrI −2.31, 3.88; Model 2: g = 0.33; 95% CrI −1.34, 1.96), light-to-moderate-to-vigorous intensity (Model 1: g = 1.22; 95% CrI −1.12, 3.87; Model 2: g = 0.25; 95% CrI −1.10, 1.62), moderate AE (Model 1: g = 0.89; 95% CrI −2.31, 2.94; Model 2: g = 0.06; 95% CrI −1.82, 1.91), moderate-to-vigorous intensity (Model 1: g = 1.82; 95% CrI −0.21, 4.21; Model 2: g = 0.45; 95% CrI −0.86, 1.72), and vigorous intensity (Model 1: g = 0.91; 95% CrI −0.63, 2.61; Model 2: g = 0.46; 95% CrI −0.42, 1.32), compared to “no treatment”. Heterogeneity was large in Model 1 (τ = 0.89) and decreased to small in Model 2 (τ = 0.30). Model 1 forest plot is presented in the Supplementary Material. See Model 2 forest plot in Figure 5.

The inclusion of both covariates (weekly frequency and number of weeks) slightly improved the precision of AE intensity estimates. Weekly frequency (w_f_ = 2.68; 95% CrI 0.68, 4.62) showed a significant positive effect on walking function, but the 95% CrI limits confidence in this result. The lower bound already suggests a large effect (0.68 per additional day), and the upper bound appears implausible. The number of weeks (w_n_ = −0.35; 95% CrI −0.8, 0.1) showed a non-significant and imprecise association with the observed effects.

Comparisons between AE intensities relied mainly on indirect evidence. Only light-to-moderate vs. vigorous had direct evidence [35], while moderate vs. vigorous presented both direct and indirect evidence [33].

Both models presented non-significant and imprecise results for pairwise comparisons, with 95% CrI, exceeding in both directions with large effect sizes (g > 0.7), preventing drawing clear conclusions about the real difference between AE intensities.

All estimated effects from both models were non-significant, with 95% CrI exceedingly wide in both directions, including large effect sizes (g > 0.7), preventing drawing clear conclusions about the effect of AE intensities; see Table 1.

A preliminary GRADE assessment of the direct networks identified “very serious” concerns for risk of bias, while heterogeneity and publication bias were rated as “not serious”, since only one study contributed to each comparison. For the indirect networks, risk of bias was judged as “very serious”, indirectness as “not serious”, and heterogeneity as “very serious” in Model 1; ranging from “not serious” to “very serious” in Model 2. Publication bias also ranged from “not serious” to “very serious”, while intransitivity was consistently judged as “not serious” in all comparisons; see Table 1 and Supplementary Material. For the mixed NMA estimates, imprecision was rated as “very serious” in both models. Final GRADE evaluation indicated “very low” certainty of evidence for all comparisons; see Table 1.

## 4. Discussion

This NMA aimed to identify the most effective AE intensity for KOA across six clinical and performance outcomes. While the analytical framework was designed to provide comparative estimates between AE intensities, the findings primarily reflect the current limitations of the available literature, rather than definitive effects of intensity.

Fifteen studies [26,27,28,29,30,31,32,33,34,35,36,37,38,39,40] were included in this review, but only two outcomes (pain intensity and walking performance) could be meta-analyzed. Neither analysis provided clear conclusions due to the wide CrI and a high risk of bias, mainly driven by: (1) low statistical power from few studies per intensity category; and (2) predominant reliance on indirect comparisons. Importantly, these results should not be interpreted as evidence of ineffectiveness, but rather as an indication that the existing literature does not yet allow reliable estimations of the real AE intensity effects. This limitation is driven not only by indirectness, but also by the high risk of bias observed across nearly all included trials, which fundamentally undermines the validity of any pooled estimate.

Given these findings, it remains highly relevant to examine AE in great detail and to assess whether exercise intensity is a determinant of its effectiveness. This relevance is supported by four key considerations: (1) recent meta-analytical evidence indicates that AE is effective, with precision and in the short term, (around 4 weeks) on pain and functional outcomes on KOA, whereas evidence for other exercise modalities remains uncertain at this time-point [7]; (2) the benefits of AE appear to be maintained at approximately 12 weeks of intervention, although presents uncertainty at longer intervention periods (around 24 weeks) [7]; (3) exercise therapy, and AE in particular, is consistently and strongly recommended by clinical guidelines in KOA [6]; (4) compared with other modalities, AE has high feasibility and scalability, making it easy to conduct increments in its intensity [41].

Consequently, distinguishing between close intensities (e.g., moderate vs. moderate-to-vigorous) remains a critical but unresolved issue. Pain outcomes may be particularly imprecise due to their multifactorial nature. Although performance outcomes might be expected to improve with intensity, higher intensities may also exacerbate symptoms by increasing risk flare-ups, as reported in some populations through the exposure to certain physical activities [42]. The scope to answer this question is limited by insufficient reporting of dropouts, and the scarce methods to mitigate attrition-related bias (e.g., data-imputation). These concerns were reflected in ROB Domain 2 with pain studies judged with “some concerns” or “high risk” of bias [28,32,34,38,39], as did all studies in walking performance. A major contributor to this assessment was the inherent lack of participant and personnel blinding in exercise-based interventions, which may have inflated perceived treatment effects, particularly in trials were subjects were compared to a usual care or no treatment condition.

To our knowledge, this is the first NMA comparing AE intensities in KOA. A previous meta-analysis including strength training found high-intensity exercise to be superior to low-intensity in pain and function, though not clinically relevant [10]. Other studies comparing AE and resistance exercise found no clear superiority [43].

Although AE alone has been considered effective for KOA, this conclusion should be interpreted with caution and extended to other exercise modalities. Limitations consistently observed across most systematic reviews include: (1) including patients not with KOA exclusively (most frequently with hip OA) [10,44,45,46]; (2) no standardized AE definitions [9,44,45]; (3) frequent combination with other interventions (physiotherapy, lifestyle recommendations, education, etc.) [9,44,45]; (4) controversial risk of bias assessment [45]; (5) low-certainty evidence by GRADE [10]; and (6) large confidence intervals leading to imprecision [9]. This review applied strict selection criteria, but few studies were identified, questioning whether AE still provides high-quality evidence as previously claimed [47].

Mechanistically, AE has multiple plausible effects on KOA. It may modulate central pain pathways by enhancing serotonin release, neuroplasticity via BDNF, and other neurotrophic agents [48,49]. It may also reduce systemic and articular inflammation, lowering cytokines implicated in KOA [50,51]. Preclinical KOA models show AE decreases IL-1β, caspase-3, and MMP-13, preventing cartilage breakdown and possibly slowing disease progression [52,53,54]. Functionally, AE can improve gait, endurance, and mobility, which may occur independently of pain changes [44]. Psychologically, AE may reduce stress, anxiety, and sleep disturbance, indirectly improving their clinical state [55,56].

Together, these findings support AE as a promising intervention for KOA. Despite uncertainty about its isolated effectiveness and optimal intensity, current evidence suggests clear potential benefits.

### 4.1. Limitations

This study presents several limitations. Firstly, few studies met the inclusion criteria, mainly due to population characteristics, insufficient detail in AE prescription, and scarcity of trials assessing isolated AE. The small number of studies reduced statistical power to detect differences between AE intensities. Secondly, there is a lack of studies directly comparing AE intensities; therefore, most evidence proceeded from indirect evidence. Thirdly, many studies presented high risk of bias.

### 4.2. Clinical Implications

AE seems to be a promising intervention for KOA; however, evidence derived from studies assessing isolated AE interventions in KOA remains limited. Current results, in addition to the support of other meta-analyses, suggest potential benefits, though it remains uncertain whether AE intensity significantly influences pain intensity or walking performance. In this context, clinical decisions should follow a pragmatic approach. Specifically, clinicians may consider the following points when prescribing AE in KOA: (1) assess the patient’s history on their clinical response to physical activity, including pain exacerbation and joint swelling; (2) based on this assessment, consider initiating AE using modalities with reduced articular loading (e.g., aquatic exercise, cycling, partial body-weight-supported exercises); (3) begin with moderate-intensity continuous exercise, and progress according to patient’s preferences, tolerance, and response to moderate-to-vigorous continuous or intervallic exercise; (4) preferably monitor AE intensity using direct physiological measures, with %HRR recommended due to its bond with resting heart rate; when direct measures are not feasible, simple and validated tools such as the talk test, Borg CR10, or Borg 6–20 scales bay be used; (5) as most interventions employ a frequency of three sessions per week, this frequency is likely to provide the greatest certainty of benefit; (6) based on evidence from existing meta-analyses, improvements in pain and physical function may be expected after approximately four weeks of intervention, with greater certainty around twelve weeks of exercise [7]; (7) AE should ideally be integrated within a multimodal management strategy, including medication control, physiotherapy, health education, and weight management.

While the optimal target intensity of AE remains undefined, this structured approach should allow clinicians to balance safety and potential benefit in the face of current evidence uncertainty. Medium-to-long-term AE programs may also provide added value by addressing common comorbidities such as overweight, hypertension, insulin resistance, and metabolic syndrome.

## 5. Conclusions

Very low certainty of evidence limits drawing precise conclusions from the effects of AE intensity on pain severity and walking performance in knee osteoarthritis. The available comparisons were characterized by concerning risk of bias, high heterogeneity, certain publication bias, and a marked imprecision of credible intervals. Overall, these findings indicate not an absence of effect, but a lack of robust primary evidence to draw precise conclusions. The present network meta-analysis therefore highlights an urgent need for high-quality, directly compared intensity categories, and adequate sample sizes. Such studies are essential to determine whether aerobic exercise intensity meaningfully influences clinical outcomes in KOA.

## Figures and Tables

**Table 1:** Summary Grading of Evidence for network meta-analysis for different intensities of aerobic exercise in KOA on pain intensity and walking performance.

Outcome	Comparison		Step Analysis	Evidence		Risk of Bias	Indirectness	Imprecision			Heterogeneity		Pub. Bias	Intransitivity	Incoherence		Cert
	Group 1	Group 2						Model	Hedges’ g (95% CrI)	Rating	Result	Rating			Z *p*-Value	Rating	
Pain int.	Light–Moderate–Vigorous	Moderate	Preliminary analysis	Direct		n.a	n.a	n.a	n.a	-	n.a	n.a	n.a	-	-	-	n.a
				Indirect	2nd order loop: LMV vs. RE vs. NT vs. M	Very serious (−2)	Not serious (0)	Model 1	−0.38 (−4.88, 4.12)	-	τ = 1.4	Very serious (−2)	Very serious (−2)	Not serious (0)	-	-	Very low
								Model 2	−0.32 (−7.49, 6.84)	-	τ = 1.4	Very serious (−2)	Very serious (−2)	Not serious (0)	-	-	
			Final analysis	Mixed	-	-	-	Model 1	−0.38 (−4.88, 4.12)	Very serious (−2)	-	-	-	-	n.a	n.a	Very low
								Model 2	−0.32 (−7.49, 6.84)	Very serious (−2)					n.a	n.a	
	Light–Moderate–Vigorous	Moderate–Vigorous	Preliminary analysis	Direct		n.a	n.a	n.a	n.a	-	n.a	n.a	n.a	n.a	-	-	n.a
				Indirect	1st order loop: LMV vs. RE vs. MV	Very serious (−2)	Not serious (0)	Model 1	−0.15 (−3.65, 3.34)	-	τ = 1.4	Very serious (−2)	Serious (−1)	Not serious (0)	-	-	Very low
								Model 2	−0.15 (−3.69, 3.38)	-	τ = 1.4	Very serious (−2)	Serious (−1)	Not serious (0)	-	-	
			Final analysis	Mixed	-	-	-	Model 1	−0.15 (−3.65, 3.34)	Very serious (−2)	-	-	-	-	n.a	n.a	Very low
								Model 2	−0.15 (−3.69, 3.38)	Very serious (−2)					n.a	n.a	
	Light–Moderate–Vigorous	Vigorous	Preliminary analysis	Direct		n.a	n.a	n.a	n.a	-	n.a	n.a	n.a	n.a	-	-	n.a
				Indirect	1st order loop: LMV vs. RE vs. V	Very serious (−2)	Not serious (0)	Model 1	0.27 (−3.32, 3.85)	-	τ = 1.4	Very serious (−2)	Serious (−1)	Not serious (0)	-	-	Very low
								Model 2	0.27 (−3.35, 3.89)	-	τ = 1.4	Very serious (−2)	Serious (−1)	Not serious (0)	-	-	
			Final analysis	Mixed	-	-	-	Model 1	0.27 (−3.32, 3.85)	Very serious (−2)	-	-	-	-	n.a	n.a	Very low
								Model 2	0.27 (−3.35, 3.89)	Very serious (−2)					n.a	n.a	
	Moderate	Moderate–Vigorous	Preliminary analysis	Direct		n.a	n.a	n.a	n.a	-	n.a	n.a	n.a	n.a	-	-	n.a
				Indirect	1st order loop: M vs. NT vs. MV	Very serious (−2)	Not serious (0)	Model 1	0.23 (−3.76, 4.21)	-	τ = 1.4	Very serious (−2)	Not serious (0)	Not serious (0)	-	-	Very low
								Model 2	0.17 (−6.67, 7.00)	-	τ = 1.4	Very serious (−2)	Not serious (0)	Not serious (0)	-	-	
			Final analysis	Mixed	-	-	-	Model 1	0.23 (−3.76, 4.21)	Very serious (−2)	-	-	-	-	n.a	n.a	Very low
								Model 2	0.17 (−6.67, 7.00)	Very serious (−2)					n.a	n.a	
	Moderate	Vigorous	Preliminary analysis	Direct		n.a	n.a	n.a	n.a	-	n.a	n.a	n.a	n.a	-	-	n.a
				Indirect	1st order loop: M vs. NT vs. V	Very serious (−2)	Not serious (0)	Model 1	0.65 (−3.42, 4.71)	-	τ = 1.4	Very serious (−2)	Serious (−1)	Not serious (0)	-	-	Very low
								Model 2	0.59 (−6.29, 7.47)	-	τ = 1.4	Very serious (−2)	Serious (−1)	Not serious (0)	-	-	
			Final analysis	Mixed	-	-	-	Model 1	0.65 (−3.42, 4.71)	Very serious (−2)	-	-	-	-	n.a	n.a	Very low
								Model 2	0.59 (−6.29, 7.47)	Very serious (−2)					n.a	n.a	
	Moderate–Vigorous	Vigorous	Preliminary analysis	Direct		n.a	n.a	n.a	n.a	-	n.a	n.a	n.a	n.a	-	-	n.a
				Indirect	1st order loop: MV vs. RE vs. V	Very serious (−2)	Not serious (0)	Model 1	0.42 (−2.49, 3.33)	-	τ = 1.4	Very serious (−2)	Serious (−1)	Not serious (0)	-	-	Very low
								Model 2	0.43 (−2.49, 3.34)	-	τ = 1.4	Very serious (−2)	Serious (−1)	Not serious (0)	-	-	
			Final analysis	Mixed	-	-	-	Model 1	0.42 (−2.49, 3.33)	Very serious (−2)	-	-	-	-	n.a	n.a	Very low
								Model 2	0.43 (−2.49, 3.34)	Very serious (−2)					n.a	n.a	
Walking perf.	Light–Moderate	Light–Moderate–Vigorous	Preliminary analysis	Direct		n.a	n.a	n.a	n.a	-	n.a	n.a	n.a	-	-	-	n.a
				Indirect	2nd order loop: LM vs. V vs. RE vs. LMV	Very serious (−2)	Not serious (0)	Model 1	0.44 (−3.42, 4.30)	-	τ = 0.89	Very serious (−2)	Serious (−1)	Not serious (0)	-	-	Low
								Model 2	−0.08 (−2.23, 2.07)	-	τ = 0.30	Not serious (0)	Not serious (0)	Not serious (0)	-	-	
			Final analysis	Mixed	-	-	-	Model 1	0.44 (−3.42, 4.30)	Very serious (−2)	-	-	-	-	n.a	n.a	Very low
								Model 2	−0.08 (−2.23, 2.07)	Very serious (−2)					n.a	n.a	
	Light–Moderate	Moderate	Preliminary analysis	Direct		n.a	n.a	n.a	n.a	-	n.a	n.a	n.a	n.a	-	-	n.a
				Indirect	1st order loop: LM vs. V vs. M	Very serious (−2)	Not serious (0)	Model 1	0.11 (−3.52, 3.74)	-	τ = 0.89	Very serious (−2)	Serious (−1)	Not serious (0)	-	-	Low
								Model 2	−0.27 (−2.74, 2.21)	-	τ = 0.30	Not serious (0)	Not serious (0)	Not serious (0)	-	-	
			Final analysis	Mixed	-	-	-	Model 1	0.11 (−3.52, 3.74)	Very serious (−2)	-	-	-	-	n.a	n.a	Very low
								Model 2	−0.27 (−2.74, 2.21)	Very serious (−2)					n.a	n.a	
	Light–Moderate	Moderate–Vigorous	Preliminary analysis	Direct		n.a	n.a	n.a	n.a	-	n.a	n.a	n.a	n.a	n.a		n.a
				Indirect	2nd order loop: LM vs. V vs. RE vs. MV	Very serious (−2)	Not serious (0)	Model 1	1.04 (−2.68, 4.76)	-	τ = 0.89	Very serious (−2)	Serious (−1)	Not serious (0)			Low
								Model 2	0.12 (−1.97, 2.22)	-	τ = 0.30	Not serious (0)	Not serious (0)	Not serious (0)			
			Final analysis	Mixed	-	-	-	Model 1	1.04 (−2.68, 4.76)	Very serious (−2)	-	-	-	-	n.a	n.a	Very low
								Model 2	0.12 (−1.97, 2.22)	Very serious (−2)					n.a	n.a	
	Light–Moderate	Vigorous	Preliminary analysis	Direct	Mangione [35]	Very serious (−2)	Not serious (0)	Model 1	−0.13 (−0.82, 0.57)	-	n.a	Not serious (0)	Not serious (0)	-	-	-	Low
								Model 2	−0.13 (−0.82, 0.57)	-	n.a	Not serious (0)	Not serious (0)				
				Indirect	n.a	n.a	n.a	n.a	n.a	-	n.a	n.a	n.a	n.a	n.a	n.a	n.a
			Final analysis	Mixed	-	-	-	Model 1	−0.13 (−3.25, 3.51)	Very serious (−2)	-	-	-	-	n.a	n.a	Very low
								Model 2	0.13 (−1.74, 1.99)	Very serious (−2)					n.a	n.a	
	Light–Moderate–Vigorous	Moderate	Preliminary analysis	Direct		n.a	n.a	n.a	n.a	-	n.a	n.a	n.a	n.a	n.a		n.a
				Indirect	2nd order loop: LMV vs. RE vs. NT vs. M	Very serious (−2)	Not serious (0)	Model 1	−0.33 (−3.51, 2.86)	-	τ = 0.89	Very serious (−2)	Very serious (−2)	Not serious (0)	-	-	Very low
								Model 2	−0.19 (−2.50, 2.13)	-	τ = 0.30	Not serious (0)	Very serious (−2)	Not serious (0)	-	-	
			Final analysis	Mixed	-	-	-	Model 1	−0.33 (−3.51, 2.86)	Very serious (−2)	-	-	-	-	n.a	n.a	Very low
								Model 2	−0.19 (−2.50, 2.13)	Very serious (−2)					n.a	n.a	
	Light–Moderate–Vigorous	Moderate–Vigorous	Preliminary analysis	Direct		n.a	n.a	n.a	n.a	-	n.a	n.a	n.a	n.a	n.a		n.a
				Indirect	1st order loop: LMV vs. RE vs. MV	Very serious (−2)	Not serious (0)	Model 1	0.60 (−2.69, 3.90)	-	τ = 0.89	Very serious (−2)	Not serious (0)	Not serious (0)	-	-	Low
								Model 2	0.20 (−1.70, 2.10)	-	τ = 0.30	Not serious (0)	Not serious (0)	Not serious (0)			
			Final analysis	Mixed	-	-	-	Model 1	0.60 (−2.69, 3.90)	Very serious (−2)	-	-	-	-	n.a	n.a	Very low
								Model 2	0.20 (−1.70, 2.10)	Very serious (−2)					n.a	n.a	
	Light–Moderate–Vigorous	Vigorous	Preliminary analysis	Direct		n.a	n.a	n.a	n.a	-	n.a	n.a	n.a	n.a	n.a		n.a
				Indirect	1st order loop: LMV vs. RE vs. V	Very serious (−2)	Not serious (0)	Model 1	−0.31 (−3.21, 2.60)	-	τ = 0.89	Very serious (−2)	Serious (−1)	Not serious (0)	-	-	Low
								Model 2	0.21 (−1.44, 1.85)	-	τ = 0.30	Not serious (0)	Not serious (0)	Not serious (0)			
			Final analysis	Mixed	-	-	-	Model 1	−0.31 (−3.21, 2.60)	Very serious (−2)	-	-	-	-	n.a	n.a	Very low
								Model 2	0.21 (−1.44, 1.85)	Very serious (−2)					n.a	n.a	
	Moderate	Moderate–Vigorous	Preliminary analysis	Direct		n.a	n.a	n.a	n.a	-	n.a	n.a	n.a	n.a	n.a		n.a
				Indirect	1st order loop: M vs. NT vs. MV	Very serious (−2)	Not serious (0)	Model 1	0.93 (−2.10, 3.95)	-	τ = 0.89	Very serious (−2)	Very serious (−2)	Not serious (0)	-	-	Very low
								Model 2	0.39 (−1.87, 2.65)	-	τ = 0.30	Not serious (0)	Very serious (−2)	Not serious (0)			
			Final analysis	Mixed	-	-	-	Model 1	0.93 (−2.10, 3.95)	Very serious (−2)	-	-	-	-	n.a	n.a	Very low
								Model 2	0.39 (−1.87, 2.65)	Very serious (−2)					n.a	n.a	
	Moderate	Vigorous	Preliminary analysis	Direct	Keogh [33]	Very serious (−2)	Not serious (0)	Model 1	−0.39 (−0.57, 1.35)	-	n.a	Not serious (0)	Not serious (0)	-	-	-	Low
								Model 2	−0.39 (−0.57, 1.35)	-	n.a	Not serious (0)	Not serious (0)	-	-	-	Low
				Indirect	1st order loop: M vs. NT vs. V	Very serious (−2)	Not serious (0)	Model 1	−0.26 (−3.97, 3.47)	-	τ = 1.41	Very serious (−2)	Serious (−1)	Not serious (0)	-	-	Very low
								Model 2	−0.28 (−3.97, 3.42)	-	τ = 1.40	Very serious (−2)	Very serious (−2)	Not serious (0)			
			Final analysis	Mixed	-	-	-	Model 1	0.02 (−2.57, 2.61)	Very serious (−2)	-	-	-	-	0.74	Not serious (0)	Very low
								Model 2	0.40 (−1.66, 2.44)	Very serious (−2)					0.73	Not serious (0)	
	Moderate–Vigorous	Vigorous	Preliminary analysis	Direct		n.a	n.a	n.a	n.a	-	n.a	n.a	n.a	n.a	n.a		n.a
				Indirect	1st order loop: MV vs. NT vs. V	Very serious (−2)	Not serious (0)	Model 1	−0.91 (−3.63, 1.81)	-	τ = 0.89	Very serious (−2)	Serious (−1)	Not serious (0)	-	-	Low
								Model 2	0.00 (−1.56, 1.57)	-	τ = 0.30	Not serious (0)	Not serious (0)	Not serious (0)			
			Final analysis	Mixed	-	-	-	Model 1	−0.91 (−3.63, 1.81)	Very serious (−2)	-	-	-	-	n.a	n.a	Very low
								Model 2	0.00 (−1.56, 1.57)	Very serious (−2)							

Cert, certainty; LM, light-to-moderate aerobic exercise; LMV, light-to-moderate-to-vigorous aerobic exercise; M, moderate aerobic exercise; MV, moderate-to-vigorous aerobic exercise; n.a., not analyzed; NT, no treatment; Pain Int., pain intensity; Pub. Bias, publication bias; RE, resistance exercise; V, vigorous aerobic exercise; Walking perf., walking performance.

## Data Availability

The original contributions presented in this study are included in the article/Supplementary Material. Further inquiries can be directed to the corresponding author.

## Supplementary Materials

The following supporting information can be downloaded at https://www.mdpi.com/article/10.3390/healthcare14040451/s1. Figure S1: methodological quality assessment with PEDro scale; Figure S2: risk of bias assessment with ROB 2.0 tool for every outcome of interest; Figure S3: forest plot of NMA Model 1 (NMA without covariates) for pain intensity; Figure S4: funnel plot of NMA Model 1 (NMA without covariates) for pain intensity; Figure S5: funnel plot of NMA Model 2 (NMA with covariates) for pain intensity; Figure S6: forest plot of NMA Model 1 (NMA without covariates) for walking performance; Figure S7: funnel plot of NMA Model 1 (NMA without covariates) for walking performance; Figure S8: funnel plot of NMA Model 2 (NMA with covariates) for walking performance; Table S1: summary table of search engines, databases and search equations; Table S2: methods of estimating intensity of cardiorespiratory exercise by the ACSM 11th edition; Table S3: summary table of included studies in the review; Table S4: data availability, extraction and estimations for meta-analyses; Text S1: description of NMA methodology; Text S2: computation of Hedges’ g and its exact variance; Text S3: Python syntax for computing Bayesian NMA, pairwise comparisons, and funnel plots.

## References

[1] Kraus V.B., Blanco F.J., Englund M., Karsdal M.A., Lohmander L.S. (2015). Call for Standardized Definitions of Osteoarthritis and Risk Stratification for Clinical Trials and Clinical Use. Osteoarthr. Cartil./OARS Osteoarthr. Res. Soc..

[2] Kellgren J.H., Lawrence J.S. (1957). Radiological Assessment of Osteo-Arthrosis. Ann. Rheum. Dis..

[3] Cui A., Li H., Wang D., Zhong J., Chen Y., Lu H. (2020). Global, Regional Prevalence, Incidence and Risk Factors of Knee Osteoarthritis in Population-Based Studies. EClinicalMedicine.

[4] Salmon J.H., Rat A.C., Achit H., Ngueyon-Sime W., Gard C., Guillemin F., Jolly D., Fautrel B. (2019). Health Resource Use and Costs of Symptomatic Knee and/or Hip Osteoarthritis. Osteoarthr. Cartil..

[5] Salmon J.H., Rat A.C., Sellam J., Michel M., Eschard J.P., Guillemin F., Jolly D., Fautrel B. (2016). Economic Impact of Lower-Limb Osteoarthritis Worldwide: A Systematic Review of Cost-of-Illness Studies. Osteoarthr. Cartil..

[6] Gibbs A.J., Gray B., Wallis J.A., Taylor N.F., Kemp J.L., Hunter D.J., Barton C.J. (2023). Recommendations for the Management of Hip and Knee Osteoarthritis: A Systematic Review of Clinical Practice Guidelines. Osteoarthr. Cartil..

[7] Yan L., Li D., Xing D., Fan Z., Du G., Jiu J., Li X., Estill J., Wang Q., Belal A.A. (2025). Comparative Efficacy and Safety of Exercise Modalities in Knee Osteoarthritis: Systematic Review and Network Meta-Analysis. BMJ.

[8] Zheng G., Qiu P., Xia R., Lin H., Ye B., Tao J., Chen L. (2019). Effect of Aerobic Exercise on Inflammatory Markers in Healthy Middle-Aged and Older Adults: A Systematic Review and Meta-Analysis of Randomized Controlled Trials. Front. Aging Neurosci..

[9] Juhl C., Christensen R., Roos E.M., Zhang W., Lund H. (2014). Impact of Exercise Type and Dose on Pain and Disability in Knee Osteoarthritis: A Systematic Review and Meta-Regression Analysis of Randomized Controlled Trials. Arthritis Rheumatol..

[10] Regnaux J.P., Lefevre-Colau M.M., Trinquart L., Nguyen C., Boutron I., Brosseau L., Ravaud P. (2015). High-Intensity versus Low-Intensity Physical Activity or Exercise in People with Hip or Knee Osteoarthritis. Cochrane Database Syst. Rev..

[11] Hutton B., Salanti G., Caldwell D.M., Chaimani A., Schmid C.H., Cameron C., Ioannidis J.P.A., Straus S., Thorlund K., Jansen J.P. (2015). The PRISMA Extension Statement for Reporting of Systematic Reviews Incorporating Network Meta-Analyses of Health Care Interventions: Checklist and Explanations. Ann. Intern. Med..

[12] Ouzzani M., Hammady H., Fedorowicz Z., Elmagarmid A. (2016). Rayyan—A Web and Mobile App for Systematic Reviews. Syst. Rev..

[13] Liguori G., Feito Y., Fountaine C.J., Roy B., American College of Sports Medicine (2022). ACSM’s Guidelines for Exercise Testing and Prescription.

[14] Borg G. (1998). Borg’s Perceived Exertion and Pain Scales.

[15] Foster C., Porcari J., Ault S., Doro K., Dubiel J., Engen M., Kolman D., Xiong S. (2018). Exercise Prescription When There Is No Exercise Test: The Talk Test. Kinesiology.

[16] Dubiel J.T. (2014). Measuring the Talk Test as a Continuous as Opposed to a Categorical Variable. Ph.D. Thesis.

[17] Morton N.A. (2009). de The PEDro Scale Is a Valid Measure of the Methodological Quality of Clinical Trials: A Demographic Study. Aust. J. Physiother..

[18] Sterne J.A.C., Savović J., Page M.J., Elbers R.G., Blencowe N.S., Boutron I., Cates C.J., Cheng H.Y., Corbett M.S., Eldridge S.M. (2019). RoB 2: A Revised Tool for Assessing Risk of Bias in Randomised Trials. BMJ.

[19] Landis J.R., Koch G.G. (1977). The Measurement of Observer Agreement for Categorical Data. Biometrics.

[20] Revelle W. (2024). Psych: Procedures for Psychological, Psychometric, and Personality Research.

[21] R Core Team (2024). R: A Language and Environment for Statistical Computing.

[22] Hedges L.V. (1982). Estimation of Effect Size from a Series of Independent Experiments. Psychol. Bull..

[23] Cohen J. (1992). A Power Primer. Psychol. Bull..

[24] Salvatier J., Wiecki T.V., Fonnesbeck C. (2016). Probabilistic Programming in Python Using PyMC3. PeerJ Comput. Sci..

[25] Izcovich A., Chu D.K., Mustafa R.A., Guyatt G., Brignardello-Petersen R. (2023). A Guide and Pragmatic Considerations for Applying GRADE to Network Meta-Analysis. BMJ.

[26] Arrieiro A., Mendonça V., Fonseca S., Santos J., Ribeiro V., Amorim M., Leopoldino A., Rodrigues Lacerda A.C. (2019). Land-Based versus Water-Based Walking Programs in Elderly Women with Knee Osteoarthritis: Preliminary Results of a Randomized Clinical Trial. Braz. J. Health Biomed. Sci..

[27] Moghadam E.B., Shojaedin S.S. (2017). The Effect of Eight Weeks Aerobic Training on Functional Indicators and Range of Motion in Active Older Men with Knee Osteoarthritis. Biol. Sci..

[28] Beckwée D., Bautmans I., Scheerlinck T., Vaes P. (2015). Exercise in Knee Osteoarthritis–Preliminary Findings: Exercise-Induced Pain and Health Status Differs between Drop-Outs and Retainers. Exp. Gerontol..

[29] Casilda-López J., Valenza M.C., Cabrera-Martos I., Díaz-Pelegrina A., Moreno-Ramírez M.P., Valenza-Demet G. (2017). Effects of a Dance-Based Aquatic Exercise Program in Obese Postmenopausal Women with Knee Osteoarthritis: A Randomized Controlled Trial. Menopause.

[30] de Almeida A.C., Aily J.B., Pedroso M.G., Gonçalves G.H., de Carvalho Felinto J., Ferrari R.J., Pastre C.M., Mattiello S.M. (2019). A Periodized Training Attenuates Thigh Intermuscular Fat and Improves Muscle Quality in Patients with Knee Osteoarthritis: Results from a Randomized Controlled Trial. Clin. Rheumatol..

[31] de Almeida A.C., Aily J.B., Pedroso M.G., Gonçalves G.H., Pastre C.M., Mattiello S.M. (2020). Reductions of Cardiovascular and Metabolic Risk Factors after a 14-Week Periodized Training Model in Patients with Knee Osteoarthritis: A Randomized Controlled Trial. Clin. Rheumatol..

[32] Ettinger W.H., Burns R., Messier S.P., Applegate W., Rejeski W.J., Morgan T., Shumaker S., Berry M.J., O’Toole M., Monu J. (1997). A Randomized Trial Comparing Aerobic Exercise and Resistance Exercise With a Health Education Program in Older Adults With Knee Osteoarthritis: The Fitness Arthritis and Seniors Trial (FAST). JAMA.

[33] Keogh J.W., Grigg J., Vertullo C.J. (2018). Is High-Intensity Interval Cycling Feasible and More Beneficial than Continuous Cycling for Knee Osteoarthritic Patients? Results of a Randomised Control Feasibility Trial. PeerJ.

[34] Lim J.-Y., Tchai E., Jang S.-N. (2010). Effectiveness of Aquatic Exercise for Obese Patients with Knee Osteoarthritis: A Randomized Controlled Trial. PM&R.

[35] Mangione K.K., McCully K., Gloviak A., Lefebvre I., Hofmann M., Craik R. (1999). The Effects of High-Intensity and Low-Intensity Cycle Ergometry in Older Adults With Knee Osteoarthritis. J. Gerontol. Ser. A Biol. Sci. Med. Sci..

[36] Messier S.P., Thompson C.D., Ettinger W.H. (1997). Effects of Long-Term Aerobic or Weight Training Regimens on Gait in an Older, Osteoarthritic Population. J. Appl. Biomech..

[37] Øiestad B.E., Årøen A., Røtterud J.H., Østerås N., Jarstad E., Grotle M., Risberg M.A. (2023). The Efficacy of Strength or Aerobic Exercise on Quality of Life and Knee Function in Patients with Knee Osteoarthritis. A Multi-Arm Randomized Controlled Trial with 1-Year Follow-Up. BMC Musculoskelet. Disord..

[38] Salacinski A.J., Krohn K., Lewis S.F., Holland M.L., Ireland K., Marchetti G. (2012). The Effects of Group Cycling on Gait and Pain-Related Disability in Individuals With Mild-to-Moderate Knee Osteoarthritis: A Randomized Controlled Trial. J. Orthop. Sports Phys. Ther..

[39] Samut G., Dinçer F., Özdemir O. (2015). The Effect of Isokinetic and Aerobic Exercises on Serum Interleukin-6 and Tumor Necrosis Factor Alpha Levels, Pain, and Functional Activity in Patients with Knee Osteoarthritis. Mod. Rheumatol..

[40] Watanabe S., Someya F. (2013). Effect of Body Weight-Supported Walking on Exercise Capacity and Walking Speed in Patients with Knee Osteoarthritis: A Randomized Controlled Trial. J. Jpn. Phys. Ther. Assoc..

[41] Pedersen M.E., Jørgensen T.S., Bandholm T., Ried-Larsen M., Bartholdy C., Runhaar J., Schiphof D., Bennell K.L., White D., King L.K. (2025). A Catalog of “Knee Friendly” Aerobic Exercises Developed for Patients With Knee Osteoarthritis: An International Patient Survey. ACR Open Rheumatol..

[42] Parry E., Ogollah R., Peat G. (2019). “Acute Flare-Ups” in Patients with, or at High Risk of, Knee Osteoarthritis: A Daily Diary Study with Case-Crossover Analysis. Osteoarthr. Cartil..

[43] Ceballos-Laita L., Lahuerta-Martín S., Carrasco-Uribarren A., Cabanillas-Barea S., Hernández-Lázaro H., Pérez-Guillén S., Jiménez-Del-Barrio S. (2023). Strength Training vs. Aerobic Training for Managing Pain and Physical Function in Patients with Knee Osteoarthritis: A Systematic Review and Meta-Analysis. Healthcare.

[44] Goh S.-L., Persson M.S.M., Stocks J., Hou Y., Welton N.J., Lin J., Hall M.C., Doherty M., Zhang W. (2019). Relative Efficacy of Different Exercises for Pain, Function, Performance and Quality of Life in Knee and Hip Osteoarthritis: Systematic Review and Network Meta-Analysis. Sports Med..

[45] Luo Y., Chen X., Gong H., Chen L., Zhang L., Li S. (2025). Efficacy of Aerobic Exercises for Knee Osteoarthritis: A Network Meta Analysis of Randomized Clinical Trials. J. Orthop. Surg. Res..

[46] Uthman O.A., Windt D.A.V.D., Jordan J.L., Dziedzic K.S., Healey E.L., Peat G.M., Foster N.E. (2014). Exercise for Lower Limb Osteoarthritis: Systematic Review Incorporating Trial Sequential Analysis and Network Meta-Analysis. Br. J. Sports Med..

[47] Fransen M., McConnell S., Harmer A.R., Van der Esch M., Simic M., Bennell K.L. (2015). Exercise for Osteoarthritis of the Knee. Cochrane Database Syst. Rev..

[48] Al-Sharman A., Khalil H., El-Salem K., Aldughmi M., Aburub A. (2019). The Effects of Aerobic Exercise on Sleep Quality Measures and Sleep-Related Biomarkers in Individuals with Multiple Sclerosis: A Pilot Randomised Controlled Trial. NeuroRehabilitation.

[49] Puts S., Liberman K., Leysen L., Forti L., Muyldermans E., Vaes P., Nijs J., Beckwée D., Bautmans I. (2023). Exercise-Induced Effects on Inflammatory Markers and Brain-Derived Neurotrophic Factor in Patients with Knee Osteoarthritis. A Systematic Review with Meta-Analysis. Exerc. Immunol. Rev..

[50] Knights A.J., Redding S.J., Maerz T. (2022). Inflammation in Osteoarthritis: The Latest Progress and Ongoing Challenges. Curr. Opin. Rheumatol..

[51] Primorac D., Molnar V., Rod E., Jeleč Ž., Čukelj F., Matišić V., Vrdoljak T., Hudetz D., Hajsok H., Borić I. (2020). Knee Osteoarthritis: A Review of Pathogenesis and State-Of-The-Art Non-Operative Therapeutic Considerations. Genes.

[52] Assis L., Milares L.P., Almeida T., Tim C., Magri A., Fernandes K.R., Medalha C., Renno A.M. (2016). Aerobic Exercise Training and Low-Level Laser Therapy Modulate Inflammatory Response and Degenerative Process in an Experimental Model of Knee Osteoarthritis in Rats. Osteoarthr. Cartil..

[53] Martins J.B., Mendonça V.A., Aguiar G.C., Fonseca S.F.D., Santos J.M.D., Tossige-Gomes R., Melo D.D.S., Oliveira M.X., Leite H.R., Camargos A.C.R. (2019). Effect of a Moderate-Intensity Aerobic Training on Joint Biomarkers and Functional Adaptations in Rats Subjected to Induced Knee Osteoarthritis. Front. Physiol..

[54] Rios J.L., Bomhof M.R., Reimer R.A., Hart D.A., Collins K.H., Herzog W. (2019). Protective Effect of Prebiotic and Exercise Intervention on Knee Health in a Rat Model of Diet-Induced Obesity. Sci. Rep..

[55] Parmelee P.A., Tighe C.A., Dautovich N.D. (2015). Sleep Disturbance in Osteoarthritis: Linkages with Pain, Disability and Depressive Symptoms. Arthritis Care Res..

[56] Xu M., Tian C., Liang S., Tong B., Wu Y., Zhou L., Nian T., Wang Y., Yang K., Li X. (2024). Comparative Efficacy of Exercise Modalities on Sleep Quality in Populations with Sleep Disorders: A Systematic Review and Network Meta-Analysis. Sleep Med. Rev..

