# Supplementary material for: How Intense Is Effective? Exploring Aerobic Exercise Intensity for Knee Osteoarthritis Through a Bayesian NetworkMeta-Analysis
Source: Healthcare (Basel). 2026 Feb 11;14(4):451. doi: 10.3390/healthcare14040451 (PMC12940493; doi:10.3390/healthcare14040451)
Supplement: Supplementary file 1 [file healthcare-14-00451-s001.zip › healthcare-4140167-supplementary.pdf]

Supplementary Material

- Table S1. Summary table of search engines, databases, and search equations.
- Table S2. Methods of estimating intensity of cardiorespiratory exercise by the ACSM 11th edition.
- Table S3. Summary table of included studies in the review.
- Table S4. Data availability, extraction, and estimations for meta-analyses
- Figure S1. Methodological quality assessment with PEDro scale.
- Figure S2. Risk of bias assessment with ROB 2.0 tool for every outcome of interest.
- Figure S3. Forest plot of NMA Model 1 (NMA without covariates) for pain intensity.
- Figure S4. Funnel plot of NMA Model 1 (NMA without covariates) for pain intensity.
- Figure S5. Funnel plot of NMA Model 2 (NMA with covariates) for pain intensity.
- Figure S6. Forest plot of NMA Model 1 (NMA without covariates) for walking performance.
- Figure S7. Funnel plot of NMA Model 1 (NMA without covariates) for walking performance.
- Figure S8. Funnel plot of NMA Model 2 (NMA with covariates) for walking performance.
- Supplementary Material Text 1. Description of NMA methodology.
- Supplementary Material Text 2. Computation of Hedges' g and its exact variance.
- Supplementary Material Text 3. Python syntax for computing Bayesian NMA, pairwise comparisons and funnel plots.

Table S1. Summary table of search engines, databases, and search equations.

Search Engine	Databases	Searches (nº)	Equation	Date	Registries (n)
PubMed	MEDLINE	Nº1	(("osteoarthritis, knee"[MeSH Terms] OR "gonarthrosis"[Title/Abstract] OR (("Knee"[Title/Abstract] OR "Tibiofemoral"[Title/Abstract] OR "Femorotibial"[Title/Abstract] OR "Knee"[MeSH Terms] OR "Knee Joint"[MeSH Terms]) AND ("Osteoarthri*"[Title/Abstract] OR "Arthrosi*"[Title/Abstract] OR "Osteoarthrosi*"[Title/Abstract] OR "Osteoarthritis"[MeSH Terms] OR (("Cartilage"[Title/Abstract] OR "Chondral"[Title/Abstract]) AND ("Defect*"[Title/Abstract] OR "Loss"[Title/Abstract] OR "Volume"[Title/Abstract] OR "Density"[Title/Abstract] OR "Thickness"[Title/Abstract] OR "Structure"[Title/Abstract] OR "Infrastructure"[Title/Abstract] OR "Surface"[Title/Abstract] OR "Degenerat*"[Title/Abstract] OR "Deform*"[Title/Abstract]))))) AND ("Circuit-Based Exercise"[MeSH Terms] OR "Endurance Training"[MeSH Terms] OR "High-Intensity Interval Training"[MeSH Terms] OR "Running"[MeSH Terms] OR "Swimming"[MeSH Terms] OR "Walking"[MeSH Terms] OR "Running"[Title/Abstract] OR "Run"[Title/Abstract] OR "Jogging"[Title/Abstract] OR "Walk*"[Title/Abstract] OR "Cycling"[Title/Abstract] OR "Swimming"[Title/Abstract] OR (("Aerobic"[Title/Abstract] OR "Endurance"[Title/Abstract] OR "Cardiovascular"[Title/Abstract] OR "Cardiopulmonar*"[Title/Abstract] OR "Cardiorespiratory"[Title/Abstract] OR "Metabolic*"[Title/Abstract] OR "Circuit"[Title/Abstract] OR "Circuit-based"[Title/Abstract] OR "Interval*"[Title/Abstract]) AND ("Exercis*"[Title/Abstract] OR "Train*"[Title/Abstract] OR "Workout*"[Title/Abstract] OR "Practice"[Title/Abstract] OR "Activit*"[Title/Abstract] OR "Conditioning"[Title/Abstract]))) AND ("Trial"[Title/Abstract] OR "randomized controlled trial"[Publication Type] OR "Randomized Controlled Trials as Topic"[MeSH Terms])) NOT ("Review"[Title] OR "Systematic review"[Title] OR "Meta analysis"[Title] OR "Metaanal*"[Title] OR "Meta anal*"[Title] OR "Review"[Publication Type] OR "Systematic review"[Publication Type] OR "Review Literature as Topic"[MeSH Terms] OR "Meta analysis"[Publication Type] OR "Meta-Analysis as Topic"[MeSH Terms])	26th Dec, 2023	1344
EBSCO	CINAHL Complete, SPORTDiscuss with full text, EBSCO eClassics Collection (EBSCOhost), OpenDissertations	Nº1	((AB "Knee osteoarthritis" OR TI "Knee osteoarthritis" OR AB "Gonarthrosis" OR TI "Gonarthrosis" OR ((AB "Knee" OR TI "Knee" OR AB "Tibiofemoral" OR TI "Tibiofemoral" OR AB "Femorotibial" OR TI "Femorotibial") AND (AB "Osteoarthri*" OR TI "Osteoarthri*" OR AB "Arthrosi*" OR TI "Arthrosi*" OR AB "Osteoarthrosi*" OR TI "Osteoarthrosi*" OR ((AB "Cartilage" OR TI "Cartilage" OR AB "Chondral" OR TI "Chondral") AND (AB "Defect*" OR TI "Defect*" OR AB "Loss" OR TI "Loss" OR AB "Volume" OR TI "Volume" OR AB "Density" OR TI "Density" OR AB "Thickness" OR TI "Thickness" OR AB "Structure" OR TI "Structure" OR AB "Infrastructure" OR TI "Infrastructure" OR AB "Surface" OR TI "Surface" OR AB "Degenerat*" OR TI "Degenerat*" OR AB "Deform*" OR TI "Deform*"))))) AND (AB "Circuit-Based Exercise" OR TI "Circuit-Based Exercise" OR AB "Endurance Training" OR TI "Endurance Training" OR AB "High-Intensity Interval Training" OR TI "High-Intensity Interval Training" OR AB "Running" OR TI "Running" OR AB "Swimming" OR TI "Swimming" OR AB "Walking" OR TI "Walking" OR AB "Running" OR TI "Running" OR AB "Run" OR TI "Run" OR AB "Jogging" OR TI	26th Dec, 2023	593

			"Jogging" OR AB "Walk*" OR TI "Walk*" OR AB "Cycling" OR TI "Cycling" OR AB "Swimming" OR TI "Swimming" OR ((AB "Aerobic" OR TI "Aerobic" OR AB "Endurance" OR TI "Endurance" OR AB "Cardiovascular" OR TI "Cardiovascular" OR AB "Cardiopulmonar*" OR TI "Cardiopulmonar*" OR AB "Cardiorespiratory" OR TI "Cardiorespiratory" OR AB "Metabolic*" OR TI "Metabolic*" OR AB "Circuit" OR TI "Circuit" OR AB "Circuit-based" OR TI "Circuit-based" OR AB "Interval*" OR TI "Interval*") AND (AB "Exercis*" OR TI "Exercis*" OR AB "Train*" OR TI "Train*" OR AB "Workout*" OR TI "Workout*" OR AB "Practice" OR TI "Practice" OR AB "Activit*" OR TI "Activit*" OR AB "Conditioning" OR TI "Conditioning"))) AND (AB "Trial" OR TI "Trial" OR AB "randomized controlled trial" OR TI "randomized controlled trial")) NOT (TI "Review" OR TI "Systematic review" OR TI "Meta analysis" OR TI "Metaanal*" OR TI "Meta anal*")		
Web of Science	Web of Science Core Collection, Current Contents Connect, Derwent Innovations Index, KCI-Korean Journal Database, ProQuest ™ Dissertations & Theses Citation Index, SciELO Citation Index	Nº1	((TS=("Knee osteoarthritis") OR TS=("Gonarthrosis") OR ((TS=("Knee") OR TS=("Tibiofemoral") OR TS=("Femorotibial")) AND (TS=("Osteoarthri*") OR TS=("Arthrosi*") OR TS=("Osteoarthrosi*") OR ((TS=("Cartilage") OR TS=("Chondral")) AND (TS=("Defect*") OR TS=("Loss") OR TS=("Volume") OR TS=("Density") OR TS=("Thickness") OR TS=("Structure") OR TS=("Infrastructure") OR TS=("Surface") OR TS=("Degenerat*") OR TS=("Deform*")))))) AND (TS=("Circuit-Based Exercise") OR TS=("Endurance Training") OR TS=("High-Intensity Interval Training") OR TS=("Running") OR TS=("Swimming") OR TS=("Walking") OR TS=("Running") OR TS=("Run") OR TS=("Jogging") OR TS=("Walk*") OR TS=("Cycling") OR TS=("Swimming") OR ((TS=("Aerobic") OR TS=("Endurance") OR TS=("Cardiovascular") OR TS=("Cardiopulmonar*") OR TS=("Cardiorespiratory") OR TS=("Metabolic*") OR TS=("Circuit") OR TS=("Circuit-based") OR TS=("Interval*")) AND (TS=("Exercis*") OR TS=("Train*") OR TS=("Workout*") OR TS=("Practice") OR TS=("Activit*") OR TS=("Conditioning")))) AND (TS=("Trial") OR TS=("randomized controlled trial"))) NOT (TI=("Review") OR TI=("Systematic review") OR TI=("Meta analysis") OR TI=("Metaanal*") OR TI=("Meta anal*"))	26th Dec, 2023	1960
ScienceDirect	ScienceDirect	Nº1	Title, abstract, keywords: (("Gonarthrosis" OR ("Knee" AND ("Osteoarthritis" OR "Arthrosis" OR "Osteoarthrosis"))) AND "Aerobic" AND ("Exercise" OR "Training") AND “Trial”) Title: NOT ("Review" OR "Systematic review" OR "Meta analysis")	26th Dec, 2023	12
		Nº2	Title, abstract, keywords: (("Gonarthrosis" OR ("Knee" AND ("Osteoarthritis" OR "Arthrosis" OR "Osteoarthrosis"))) AND "Endurance" AND ("Exercise" OR "Training") AND “Trial”) Title: NOT ("Review" OR "Systematic review" OR "Meta analysis")	26th Dec, 2023	3
		Nº3	Title, abstract, keywords: (("Gonarthrosis" OR ("Knee" AND ("Osteoarthritis" OR "Arthrosis" OR "Osteoarthrosis"))) AND "Cardiovascular" AND ("Exercise" OR "Training") AND “Trial”) Title: NOT ("Review" OR "Systematic review" OR "Meta analysis")	26th Dec, 2023	4
		Nº4	Title, abstract, keywords: (("Gonarthrosis" OR ("Knee" AND ("Osteoarthritis" OR "Arthrosis" OR "Osteoarthrosis"))) AND "Cardiopulmonar" AND ("Exercise" OR "Training") AND “Trial”) Title: NOT ("Review" OR "Systematic review" OR "Meta analysis")	26th Dec, 2023	0
		Nº5	Title, abstract, keywords: (("Gonarthrosis" OR ("Knee" AND ("Osteoarthritis" OR "Arthrosis" OR "Osteoarthrosis"))) AND "Cardiorespiratory" AND ("Exercise" OR "Training") AND “Trial”) Title: NOT ("Review" OR "Systematic review" OR "Meta analysis")	26th Dec, 2023	3
		Nº6	Title, abstract, keywords: (("Gonarthrosis" OR ("Knee" AND ("Osteoarthritis" OR "Arthrosis" OR "Osteoarthrosis"))) AND "Metabolic" AND ("Exercise" OR "Training") AND “Trial”)	26th Dec, 2023	4

			Title: NOT ("Review" OR "Systematic review" OR "Meta analysis")		
		Nº7	Title, abstract, keywords: (("Gonarthrosis" OR ("Knee" AND ("Osteoarthritis" OR "Arthrosis" OR "Osteoarthrosis"))) AND "Circuit" AND ("Exercise" OR "Training") AND "Trial") Title: NOT ("Review" OR "Systematic review" OR "Meta analysis")	26th Dec, 2023	2
		Nº8	Title, abstract, keywords: (("Gonarthrosis" OR ("Knee" AND ("Osteoarthritis" OR "Arthrosis" OR "Osteoarthrosis"))) AND "Interval" AND ("Exercise" OR "Training") AND "Trial") Title: NOT ("Review" OR "Systematic review" OR "Meta analysis")	26th Dec, 2023	40
Scopus	Scopus	Nº1	((TITLE-ABS ("Knee osteoarthritis") OR TITLE-ABS ("Gonarthrosis") OR ((TITLE-ABS ("Knee") OR TITLE-ABS ("Tibiofemoral") OR TITLE-ABS ("Femorotibial")) AND (TITLE-ABS ("Osteoarthri*") OR TITLE-ABS ("Arthrosi*") OR TITLE-ABS ("Osteoarthrosi*") OR ((TITLE-ABS ("Cartilage") OR TITLE-ABS ("Chondral")) AND (TITLE-ABS ("Defect*") OR TITLE-ABS ("Loss") OR TITLE-ABS ("Volume") OR TITLE-ABS ("Density") OR TITLE-ABS ("Thickness") OR TITLE-ABS ("Structure") OR TITLE-ABS ("Infrastructure") OR TITLE-ABS ("Surface") OR TITLE-ABS ("Degenerat*") OR TITLE-ABS ("Deform*")))))) AND (TITLE-ABS ("Circuit-Based Exercise") OR TITLE-ABS ("Endurance Training") OR TITLE-ABS ("High-Intensity Interval Training") OR TITLE-ABS ("Running") OR TITLE-ABS ("Swimming") OR TITLE-ABS ("Walking") OR TITLE-ABS ("Running") OR TITLE-ABS ("Run") OR TITLE-ABS ("Jogging") OR TITLE-ABS ("Walk*") OR TITLE-ABS ("Cycling") OR TITLE-ABS ("Swimming") OR ((TITLE-ABS ("Aerobic") OR TITLE-ABS ("Endurance") OR TITLE-ABS ("Cardiovascular") OR TITLE-ABS ("Cardiopulmonar*") OR TITLE-ABS ("Cardiorespiratory") OR TITLE-ABS ("Metabolic*") OR TITLE-ABS ("Circuit") OR TITLE-ABS ("Circuit-based") OR TITLE-ABS ("Interval*")) AND (TITLE-ABS ("Exercis*") OR TITLE-ABS ("Train*") OR TITLE-ABS ("Workout*") OR TITLE-ABS ("Practice") OR TITLE-ABS ("Activit*") OR TITLE-ABS ("Conditioning")))) AND (TITLE-ABS ("Trial") OR TITLE-ABS ("randomized controlled trial"))) AND NOT (TITLE ("Review") OR TITLE ("Systematic review") OR TITLE ("Meta analysis") OR TITLE ("Metaanal*") OR TITLE ("Meta anal*"))	26th Dec, 2023	1186
SciELO	SciELO	Nº1	((ab:("Knee osteoarthritis")) OR (ti:("Knee osteoarthritis")) OR (ab:("Gonarthrosis")) OR (ti:("Gonarthrosis")) OR (((ab:("Knee")) OR (ti:("Knee"))) AND ((ab:("Osteoarthritis")) OR (ti:("Osteoarthritis")) OR (ab:("Arthrosis")) OR (ti:("Arthrosis")) OR (ab:("Osteoarthrosis")) OR (ti:("Osteoarthrosis"))))) AND ((ab:("Running")) OR (ti:("Running")) OR (ab:("Swimming")) OR (ti:("Swimming")) OR (ab:("Walking")) OR (ti:("Walking")) OR (ab:("Running")) OR (ti:("Running")) OR (ab:("Run")) OR (ti:("Run")) OR (ab:("Jogging")) OR (ti:("Jogging")) OR (ab:("Walking")) OR (ti:("Walking")) OR (ab:("Cycling")) OR (ti:("Cycling")) OR (ab:("Swimming")) OR (ti:("Swimming")) OR (((ab:("Aerobic")) OR (ti:("Aerobic")) OR (ab:("Endurance")) OR (ti:("Endurance")) OR (ab:("Cardiovascular")) OR (ti:("Cardiovascular")) OR (ab:("Cardiopulmonar")) OR (ti:("Cardiopulmonar")) OR (ab:("Cardiorespiratory")) OR (ti:("Cardiorespiratory")) OR (ab:("Metabolic")) OR (ti:("Metabolic")) OR (ab:("Circuit")) OR (ti:("Circuit")) OR (ab:("Interval")) OR (ti:("Interval"))) AND ((ab:("Exercise")) OR (ti:("Exercise")) OR (ab:("Training")) OR (ti:("Training")) OR (ab:("Workout")) OR (ti:("Workout)) OR (ab:("Practice")) OR (ti:("Practice")) OR (ab:("Activity")) OR (ti:("Activity")) OR (ab:("Conditioning")) OR (ti:("Conditioning"))))) AND (ab:("Trial")) OR (ti:("Trial"))	26th Dec, 2023	3

Google Scholar	-	№1	(("Gonarthrosis" OR ("Knee" + ("Osteoarthritis" OR "Arthrosis" OR "Osteoarthrosis"))) + ("Running" OR "Swimming" OR "Walking" OR "Running" OR "Run" OR "Jogging" OR "Walking" OR "Cycling" OR "Swimming" OR "Aerobic" OR "Endurance" OR "Cardiovascular" OR "Cardiopulmonar" OR "Cardiorespiratory" OR "Metabolic" OR "Circuit") + ("Exercise" OR "Training" OR "Workout" OR "Practice" OR "Activity" OR "Conditioning") + "Trial") -intitle:"review" -intitle:"systematic review" -intitle:"meta analysis"	26th Dec, 2023	980

Table S2. Methods of estimating intensity of cardiorespiratory exercise by the ACSM 11th edition.

**Relative intensity**				**Intensity (%O_2max) Relative to maximal exercise capacity in met**				**Abs. Int.**	**Abs. Int. (met) by age**		
**Intensity**	%HRR or %VO_2R	%HR max	%VO_2 max	Perceived Exertion (Rating on 6-20 RPE Scale)	20 METs %VO_2 max	10 METs %VO_2 max	5 METs %VO_2 max	(METs)	Young (20-39 yr)	Middle Age (40-64 yr)	Older (≥65 yr)
**Very light**	<30	<57	<37	Very light (RPE <9)	<34	<37	<44	<2.0	<2.4	<2.0	<1.6
**Light**	30-39	57-63	37-45	Very light to fairly light (RPE 9-11)	34-42	37-45	44-51	2.0-2.9	2.4-4.7	3.0-5.9	1.6-3.1
**Moderate**	40-59	64-76	46-63	Fairly light to somewhat hard (RPE 12-13)	43-61	46-63	52-67	3.0-5.9	4.8-7.1	5.9-9.3	4.7
**Vigorous**	60-89	77-95	64-90	Somewhat hard to very hard (RPE 14-17)	62-90	64-90	68-91	6.0-8.7	7.2-10.1	8.0-10.4	6.7
**Near-maximal to maximal**	≥90	≥96	≥91	Very hard (RPE ≥18)	≥91	≥91	≥92	≥8.8	≥10.2	≥8.5	≥6.8

Table S3. Summary table of included studies in the review.

Study	Population	Groups	Prescription parameters and monitorization	Outcome measures and instruments	Results (post-immediate)	
					Narrative	Effect direction
**Arrieiro et al., 2019 [26]** *Parallel RCT*	**KOA diagnostic criteria:** Clinical and radiological according to ACR *(only radiological reference)* **Presence of pain:** Probable but unclear	**Vigorous intensity AE** (n_{allo}=8, n_{an}=8): Age: 68.6 ± 6; F (n): 8; BMI: 28.7 ± 5.39; Diabetes: n/a; Hypertension: n/a **Vigorous intensity AE** (n_{allo}=8, n_{an}=8): Age: 67 ± 3; F(n): 8; BMI: 27.56 ± 2.05; Diabetes: n/a; Hypertension: n/a	**Continuous Land-based walking: Condit. Intensity:** Moderate (70-75% HR_{max}) **Final intensity:** Vigorous (75-80%, 75-80% and 80-85% HR_{max}) **Monit. Device:** Polar, model F4 **Volume:** Total duration (45, 50, 55, 60 and 65 min); Stimuli duration (35, 40, 45, 50 and 55 min); 3 sess/week for 12 weeks<br>**Continuous Walter-based walking: Condit. Intensity:** Moderate (70-75% HR_{max}) **Final intensity:** Vigorous (75-80%, 75-80% and 80-85% HR_{max}) **Monit. Device:** Polar, model F4 **Volume:** Total duration (45, 50, 55, 60 and 65 min); Stimuli duration (35, 40, 45, 50 and 55 min); 3 sess/week for 12 weeks	Pain intensity (WOMAC pain subscale)	No relevant differences between groups were found	Vig AE Water-based ≈ Vig AE Land-based
				Walking performance (6MWT)	No relevant differences between groups were found	Vig AE Water-based ≈ Vig AE Land-based
				Stiffness (WOMAC-Stiffness)	No relevant differences between groups were found	Vig AE Water-based ≈ Vig AE Land-based
**Bavardi Moghadam & Shojaedin 2017 [27]** *Parallel RCT*	**KOA diagnostic criteria:** Clinical and radiological criteria *(no followed guidelines)* **Presence of pain:** Yes, pain during the previous 6 months	**Moderate-to-Vigorous AE** (n_{allo}=10, n_{an}=9): Age: 64.66 ± 3.46; F (n=10); BMI: 24.93 ±0.92; Diabetes n/a; Hypertension: n/a **No treatment** (n_{allo}=10, n_{an}=9): Age: 68.77 ± 2.99; F (n=10); BMI: 25.39 ± 1.87; Diabetes n/a; Hypertension: n/a	**Continuous walking: Condit. Intensity:** 60%. Light **Final intensity:** Moderate-to-Vigorous; 65-75% HR_{max} **Monit. Device:** Pulsometer (No model info.) **Volume:** Total duration (No info.); Stimuli duration (20-30min); 3 sess/week for 8 weeks<br>**No treatment: Volume:** 8 weeks	Walking performance (6MWT)	There was a significant improvement in the walking group compared to the control group	Mod-Vig AE > No Treatment
				Function in sit-to-stand and linear walk (chair-to-stand and walk 15.2m)	There was a significant improvement in the walking group compared to the control group	Mod-Vig AE < No Treatment
**Beckwee et al., 2015 [28]** *Parallel RCT*	**KOA diagnostic criteria:** Clinical and radiological according to ACR (Altman, 1995) **Presence of pain:** Yes, knee pain in the previous 30 days	**Vigorous AE** (n_{allo}=19, n_{an}=17): Age (mdn[IQR]): 60[10]; F (n): 8; BMI (mdn[IQR]): 27.9[5.2]; Diabetes: n/a; Hypertension (mdn[IQR] n): none **Resistance exercise** (n_{allo}=19, n_{an}=15): Age (mdn[IQR]): 61[10]; F (n): 13; BMI (mdn[IQR]):	**Continuous walking: Condit. Intensity:** No **Final intensity:** Vigorous (14-17 Borg) **Monit. Device:** Borg scale (6-20 points) **Volume:** Total duration (No info.); Stimuli duration (40 min); 3 sess/week for 18 weeks<br>**Resistance exercise:**	Pain intensity during exercise (NRS post-pre)	No relevant differences between groups were found	Vig AE ≈ Resistance exercise
				Maximum pain intensity perceived (NRS-max24)	No relevant differences between groups were found	Vig AE ≈ Resistance exercise

		27.1[7.9]; Diabetes: n/a; Hypertension (mdn[IQR] n): 1[5]	**Volume:** Total duration (45 min); Stimuli duration (No info.); 3 sess/week; 18 weeks			
**Casilda-López et al., 2017 [29]** *Parallel RCT*	**KOA diagnostic criteria:** Clinical *(no guidelines followed)* **Presence of pain:** Probable but unclear	**Vigorous intensity AE** (n_{allo}=17, n_{an}=17): Age: 66 ± 6.35; F (n=17); BMI: 33.65 ± 3.04; Diabetes: n/a; Hypertension: n/a **Vigorous intensity AE** (n_{allo}=17, n_{an}=17): Age: 65.62 ± 7.15; F (n=17); BMI: 31.69 ± 2.44; Diabetes: n/a; Hypertension: n/a	**Continuous aquatic global exercises: Condit. Intensity:** No **Final intensity:** Vigorous (4-6 Borg CR-10) **Monit. Device:** Borg scale CR-10 **Volume:** Total duration (45 min); Stimuli duration (21 min); 3 sess/week for 8 weeks **Intervallic aquatic dance: Condit. Intensity:** No **Final intensity:** Vigorous (4-6 Borg CR-10) **Monit. Device:** Borg scale CR-10 **Volume:** Total duration (45 min); Stimuli duration (21 min); 3 sess/week for 8 weeks	Pain (WOMAC-Pain)	Reduction in aquatic dance group compared to the global aquatic exercise group	Vig AE Aquatic dance < Vig AE Global exercise
				Walking performance (6MWT (m))	An increase was found in aquatic dance group compared to the global aquatic exercise group	Vig AE Aquatic dance > Vig AE Global exercise
				Knee stiffness (WOMAC-Stiffness)	No differences between groups were found	Vig AE Aquatic dance ≈ Vig AE Global exercise
				Disability associated with KOA (WOMAC total score)	Reduction in aquatic dance group compared to the global aquatic exercise group	Vig AE Aquatic dance < Vig AE Global exercise
**De Almeida., 2019 [30]** *Parallel RCT*	**KOA diagnostic criteria:** Clinical and radiological according to ACR (Altman, 1986) **Presence of pain:** Yes, knee pain the previous week of ≥ 4 points on VAS (0-10)	**Light-to-moderate-to-vigorous AE** (n_{allo}=22, n_{an}=20): Age: 55.6 ± 5.3; F (n=15); BMI: 26 ± 3.08; Diabetes: n/a; Hypertension: n/a **Resistance training** (n_{allo}=22, n_{an}=21): Age: 55.2 ± 7.4; F (n=16); BMI: 26 ± 3.14; Diabetes: n/a; Hypertension: n/a **Educational protocol** (n_{allo}=22, n_{an}=20): Age: 53.8 ± 7.7; F (n=16); BMI: 27 ± 2.7; Diabetes: n/a; Hypertension: n/a	**Intervallic full-body circuit: Condit. Intensity:** Very light (<54% HR_{max} / 6-10 Borg) **Final intensity:** Light-to-moderate-to-vigorous (<54% HR_{max} / 6-10 Borg progressed to 55-69% HR_{max} / 11-14 Borg, progressed to >70% HR_{max} / 15-20 Borg) **Monit. Device:** No info for HR, Borg scale (6-20 points) **Volume:** Total duration (30-45 min); Stimuli duration (20-35 min); 3 sess/week for 14 weeks **Resistance exercise: Volume:** Total duration (60 min); Stimuli duration (50 min); 3 sess/week for 14 weeks **Education protocol: Volume:** Total duration (60 min); Stimuli duration (No info.); 0.57 sess/week (2 sess/month) for 14 weeks	Pain intensity (VAS)	Greater reduction in the circuit training group compared to the educational group No differences between aerobic and resistance group	Light-Mod-Vig AE < Educational group Light-Mod-Vig AE ≈ Resistance exercise
**De Almeida et al., 2020 [31]**	**KOA diagnostic criteria:** Clinical and radiological	**Light-to-moderate-to-vigorous AE** (n_{allo}=22, n_{an}=20): Age: 55.6 ± 5.3; F (n=15); BMI: 26 ±	**Intervallic full-body circuit: Condit. Intensity:** Very light (<54% HR_{max} / 6-10 Borg).	Pain intensity (WOMAC-Pain)	Greater reduction in the circuit training group compared to the educational group	Light-Mod-Vig AE < Educational group

Parallel RCT<br>*Same sample as Almeida et al. 2019*	according to ACR (Altman, 1986)<br>**Presence of pain:** Yes, knee pain the previous week of ≥ 4 points on VAS (0-10)	3.08; Diabetes: n/a; Hypertension: n/a<br>**Resistance training** (n_{allo}=22, n_{an}=21): Age: 55.2 ± 7.4; F (n=16); BMI: 26 ± 3.14; Diabetes: n/a; Hypertension: n/a<br>**Educational protocol** (n_{allo}=22, n_{an}=20): Age: 53.8 ± 7.7; F (n=16); BMI: 27 ± 2.7; Diabetes: n/a; Hypertension: n/a	**Final intensity:** Light-to-moderate-to-vigorous (<54% HR_{max} / 6-10 Borg progressed to 55-69% HR_{max} / 11-14 Borg, progressed to >70% HR_{max} / 15-20 Borg)<br>**Monit. Device:** No info for HR, Borg scale (6-20 points)<br>**Volume:** Total duration (30-45 min); Stimuli duration (20-35 min); 3 sess/week for 14 weeks<br>**Resistance exercise:** **Volume:** Total duration (60 min); Stimuli duration (50 min); 3 sess/week for 14 weeks<br>**Education protocol:** **Volume:** Total duration (60 min); Stimuli duration (No info.); 0.57 sess/week (2 sess/month) for 14 weeks		No differences between aerobic and resistance group	Light-Mod-Vig AE ≈ Resistance exercise
				Walking performance (40mWT (m/s))	No differences between groups were found after treatment	Light-Mod-Vig AE ≈ Educational group
					No differences between aerobic and resistance group	Light-Mod-Vig AE ≈ Resistance exercise
				Function in sit-to-stand (30sSTS)	Greater increase in the circuit training group compared to the educational group	Light-Mod-Vig AE > Educational group
					No differences between aerobic and resistance group	Light-Mod-Vig AE ≈ Resistance exercise
				Stiffness (WOMAC-Stiffness)	Greater reduction in the circuit training group compared to the educational group	Light-Mod-Vig AE < Educational group
					No differences between aerobic and resistance group	Light-Mod-Vig AE ≈ Resistance exercise
				Disability associated with KOA (WOMAC-Total)	Greater reduction in the circuit training group compared to the educational group	Light-Mod-Vig AE < Educational group
					No differences between aerobic and resistance group	Light-Mod-Vig AE ≈ Resistance exercise
**Ettinger et al., 1997 [32]**<br>*Parallel RCT*	**KOA diagnostic criteria:** Clinical and radiological (*no guidelines followed*)<br>**Presence of pain:** Yes, pain on most days and pain during activities	**Moderate-to-Vigorous AE** (n_{allo}=144, n_{an}=117): Age: 69 ± 6; F (n=99); BMI: n/a; Obesity (n=72); Diabetes (n=10); Hypertension (n=58)<br>**Resistance exercise** (n_{allo}=146, n_{an}=120): Age: 68 ± 6; F (n=107); BMI: n/a; Obesity (n=72); Diabetes (n=14); Hypertension (n=61)<br>**Education protocol** (n_{allo}=149, n_{an}=127): Age: 69 ± 6; F (n=102); BMI: n/a; Obesity (n=87); Diabetes (n=16); Hypertension (n=75)	**Continuous walking:** **Condit. Intensity:** No<br>**Final intensity:** Moderate-to-Vigorous (50-70% HRR)<br>**Monit. Device:** No info.<br>**Volume:** Total duration (60min); Stimuli duration (40 min); 3 sess/week for 79 weeks<br>**Resistance exercise:** **Volume:** Total duration (60 min); Stimuli duration (40 min); 3 sess/week; 79 weeks<br>**Education protocol:** **Volume:** Total duration (110 min); Stimuli duration (No info.); 0.25 sess/week (1 sess/month); 79 weeks	Pain intensity-during walking and transfers (1-6 scale)	Aerobic group showed less pain after intervention compared to education group	Mod-Vig AE < Education group
					No post-intervention comparisons between aerobic and resistance groups reported	No info.
				Walking performance (6MWT)	Aerobic group walked greater distance after intervention compared to education group	Mod-Vig AE > Education group
					No post-intervention comparisons between aerobic and resistance groups reported	No info.
**Keogh et al., 2018 [33]**	**KOA diagnostic criteria:** No	**Vigorous AE** (n_{allo}=15, n_{an}=9):	**Intervallic cycling:** **Condit. Intensity:** No	Walking performance (normal walk	No differences were found after treatment between groups	Vig AE ≈ Mod AE

Parallel RCT	info concerning if clinical and/or radiological *(conducted by a surgeon, and no guidelines followed)* **Presence of pain:** Unclear	Age: 59.1 ± 6.7; F (n=6); BMI: 27.0 ± 4; Diabetes n/a; Hypertension: n/a **Moderate AE** (n_{allo}=12, n_{an}=8): Age: 66.1 ± 8.8; F (n=7); BMI: 28.2 ±6.9; Diabetes: n/a; Hypertension: n/a	**Final intensity:** Vigorous *("an intensity in which you felt it was quite difficult to complete sentences during the exercise")* **Monit. Device:** Talk test. **Volume:** Total duration (25 min); Stimuli duration (11.25 min, involving 5 intervals of 45 s high intensity bouts and 90 s recovery); 4 sess/week for 8 weeks	speed in 3.66 m)		
				Function in sit-to-stand (30s chair-to-stand)	No differences were found after treatment between groups	Vig AE ≈ Mod AE
				Function in sit-to-stand and walking (TUG)	Significant reduction after treatment in the HIIT group compared to MICT	Vig AE < Mod AE
			**Continuous cycling: Condit. Intensity:** No **Final intensity:** Moderate *("an intensity in which you are able to speak in complete sentences during exercise")* **Monit. Device:** Talk test. **Volume:** Total duration (25 min); Stimuli duration (20 min); 4 sess/week for 8 weeks	Disability associated with KOA (WOMAC-total)	No differences were found after treatment between groups	Vig AE ≈ Mod AE
**Lim et al., 2010 [34]** *Parallel RCT*	**KOA diagnostic criteria:** Radiological according to KL criteria *(grades ≥2)* **Presence of pain:** Probable but unclear	**Moderate-to-Vigorous AE** (n_{allo}=26, n_{an}=24): Age: 65.7 ± 8.9; F (n=23); BMI: 27.9 ± 1.5; Diabetes n/a; Hypertension: n/a **Resistance, Aerobic, and Stretching exercises** (n_{allo}=25, n_{an}=22): Age: 67.7 ± 7.7; F (n=21); BMI: 27.6 ± 7.7; Diabetes n/a; Hypertension: n/a **Resistance exercise and Behavioral-lifestyle modification** (n_{allo}=24, n_{an}=20): Age: 63.3 ± 5.3; F (n=21); BMI: 27.7 ± 2; Diabetes n/a; Hypertension: n/a	**Continuous aquatic exercise: Condit. Intensity:** No **Final intensity:** Moderate-to-Vigorous (≥65% HR_{max}) **Monit. Device:** No info. **Volume:** Total duration (40min); Stimuli duration (30 min); 3 sess/week for 8 weeks **Resistance, Aerobic, and Stretching exercises: Volume:** Total duration (40 min); Stimuli duration (30 min); 3sess/week for 8 weeks **Resistance exercise and Behavioral-lifestyle modification: Volume:** Total duration (No info); Stimuli duration (No info); No info for sess/week; 8 weeks	Pain intensity (BPI scale)	No differences between aerobic and the group combining resistance, aerobic and stretching exercises	Mod-Vig AE ≈ Resistance, aerobic and stretching
					No differences between aerobic and the group combining resistance, and behavioral modifications	Mod-Vig AE ≈ Resistance exercise and behavioral
				Disability associated with KOA (WOMAC-total)	Greater decrease in disability in the water based aerobic exercise compared to resistance and behavioral after treatment	Mod-Vig AE < Resistance exercise and behavioral
					No differences after treatment were found between aquatic aerobic exercise and resistance, aerobic and stretching	Mod-Vig AE ≈ Resistance, aerobic and stretching
**Mangione et al., 1999 [35]** *Parallel RCT*	**KOA diagnostic criteria:** Clinical and radiological according to ACR (Altman, 1986) **Presence of pain:** Yes	**Vigorous AE** (n_{allo}=19, n_{an}*): Age: 71.1 ± 7.7; F (n=14); BMI: 29.63 ± 5.18; Diabetes n/a; Hypertension: n/a **Light-to-moderate AE** (n_{allo}=20, n_{an}*): Age: 71.0 ± 6.2; F (n=12); BMI: 29.08 ± 5.07; Diabetes n/a; Hypertension: n/a	**Continuous stationary cycling: Condit. Intensity:** No **Final intensity:** Vigorous (70% HRR) **Monit. Device:** Polar Electro, Inc., Port Washington, NY **Volume:** Total duration (60min); Stimuli duration (25 min); 3 sess/week for 10 weeks	Walking performance (6MWT, normal walk speed in 3.87m and fast walk speed in 3.87 m)	No differences found between groups after intervention	Vig AE ≈ Light-mod AE
				Function in sit-to-stand (10 chair-to-stand)	No differences found between groups after intervention	Vig AE ≈ Light-mod

			**Continuous Stationary cycling:** **Condit. Intensity:** No **Final intensity:** Light-to-Moderate (40% HRR) **Monit. Device:** Polar Electro, Inc., Port Washington, NY **Volume:** Total duration (60 min); Stimuli duration (25 min); 3 sess/week for 10 weeks			
**Messier et al., 1997 [36]** *Sub-sample of Ettinger 1997* *Parallel RCT*	**KOA diagnostic criteria:** Clinical and radiological according to ACR (Altman, 1986) **Presence of pain:** Yes, pain on most days of the month	**Moderate-to-Vigorous AE** (n_{allo}=33, n_{an}=33): Age: 70.3 ± 1.3; F (n=27); BMI: 31.4 ±1.0; Diabetes n/a; Hypertension: n/a **Resistance exercise** (n_{allo}=34, n_{an}=34): Age: 67.2 ± 0.9; F (n=23); BMI: 30.1 ± 0.9; Diabetes n/a; Hypertension: n/a **Education protocol** (n_{allo}=36, n_{an}=36): Age: 69.2 ± 1.0; F (n=28); BMI: 32.5 ± 0.9; Diabetes n/a; Hypertension: n/a	**Continuous walking:** **Condit. Intensity:** No **Final intensity:** Moderate-to-Vigorous (50-85% HRR) **Monit. Device:** No info. **Volume:** Total duration (50 min); Stimuli duration (40 min); 3 sess/week for 79 weeks† **Resistance exercise:** **Volume:** Total duration (No info.); Stimuli duration (No info.) 3sess/week; 79 weeks† **Education protocol:** **Volume:** Total duration (60 min); Stimuli duration (No info.); 0.5 phone call/week; 79 weeks†	Pain intensity during ambulation and during transfers (1-6 scale)	No differences found between aerobic and education<br>No differences found between aerobic and resistance exercise	Mod-Vig AE ≈ Education<br>Mod-Vig AE ≈ Resistance
				Walking performance (22mWT) (cm/s)	There was a significant increase in walking speed in the aerobic group compared to the education group<br>No differences found between aerobic and resistance group	Mod-Vig AE > Education<br>Mod-Vig AE ≈ Resistance
**Øiestad et al., 2023 [37]** *Parallel RCT*	**KOA diagnostic criteria:** Clinical and radiological according to ACR (Altman, 1986; Kellgren, 1957) **Presence of pain:** Yes, pain on most days of the month	**Moderate-to-Vigorous AE** (n_{allo}=55, n_{an}=42): Age: 57.3 ± 7.1; F (n=28); BMI: 29.4 ± 4.4; Diabetes n/a; Hypertension: n/a **Resistance exercise** (n_{allo}=57, n_{an}=49): Age: 57.6 ± 6.6; F (n=30); BMI: 28.9 ± 4.3; Diabetes n/a; Hypertension: n/a **No treatment** (n_{allo}=56, n_{an}=45): Age: 57.8 ± 7.4; F (n=24); BMI: 28.4 ± 4.1; Diabetes n/a; Hypertension: n/a	**Continuous stationery cycling:** **Condit. Intensity:** Yes (No info.) **Final intensity:** Moderate-to-Vigorous; 70-80% HR_{max} **Monit. Device:** No info. **Volume:** Total duration (45 min); Stimuli duration (30 min); 2 to 3 sess/week for 12 weeks § **Resistance and balance exercise:** **Volume:** Total duration (No info.); Stimuli duration (No info.); 2 to 3sess/week; 12 weeks § **No treatment:** **Volume:** 12 weeks	Pain intensity (NRS average perceived last 2 weeks)	No differences found between aerobic and no treatment group<br>No differences found between aerobic and resistance group	Mod-Vig AE ≈ No Treatment<br>Mod-Vig AE ≈ Resistance
**Salacinski et al., 2012 [38]** *Parallel RCT*	**KOA diagnostic criteria:** Clinical and radiological according to KL criteria *(grades I-III)*	**Moderate AE** (n_{allo}=19, n_{an}=19): Age: 55.1 ± 10.5; F (n=15); BMI: 22.4 ±3.3; Diabetes n/a; Hypertension: n/a **No treatment** (n_{allo}=18, n_{an}=18):	**Continuous stationary cycling:** **Condit. Intensity:** No **Final intensity:** Moderate-to-Vigorous; 70-75% HR_{max} **Monit. Device:** Polar Electro Inc, Lake Success, NY	Pain intensity (VAS at rest and after 6 min normal walking)	No differences between groups after intervention for pain at rest<br>Aerobic group showed greater reduction than the no treatment	Mod-Vig AE ≈ No Treatment<br>Mod-Vig AE < No Treatment

	**Presence of pain:** Yes, pain on the previous month	Age: 60.6 ± 8.4; F (n=12); BMI: 25.7 ± 6.3; Diabetes n/a; Hypertension: n/a	**Volume:** Total duration (40 progressed to 60 min); Stimuli duration (No info.); 2 sess/week for 12 weeks<br><br>**No treatment:** **Volume:** 12 weeks		group after 6 min of normal walking	
				Walking performance (normal walk speed in 3.66m, maximal walk speed at 3.66m)	Aerobic group showed greater normal walk speed than the no treatment group	Mod-Vig AE > No Treatment
					No differences found between groups for maximal walk speed	Mod-Vig AE ≈ No Treatment
				Stiffness (WOMAC-Stiffness)	Aerobic group showed stiffness than the no treatment group	Mod-Vig AE < Control
				Disability associated with KOA (WOMAC-Total)	No differences found between aerobic and no treatment group	Mod-Vig AE ≈ Control
**Samut et al., 2015 [39]** *Parallel RCT*	**KOA diagnostic criteria:** Clinical and radiological criteria according to ACR *(No specific reference)*<br><br>**Presence of pain:** Probable but unclear	**Vigorous AE** (n_{allo}=14, n_{an}=14): Age: 57.57 ± 5.79; F: n/a; BMI: 33.94 ± 7.33; Diabetes n/a; Hypertension: n/a<br>**Resistance exercise** (n_{allo}=15, n_{an}=13): Age: 62.46 ± 7.71; F: n/a; BMI: 30.54 ± 4.45; Diabetes n/a; Hypertension: n/a<br>**No treatment** (n_{allo}=13, n_{an}=13): Age: 60.92 ± 8.85; F: n/a; BMI: 30.36 ± 5.67; Diabetes n/a; Hypertension: n/a	**Vigorous stationary cycling:** **Condit. Intensity:** Vigorous; 65-70% HRR **Final intensity:** Vigorous; 70-75% HRR **Monit. Device:** No info. **Volume:** Total duration (No info.); Stimuli duration (No info.); 3 sess/week for 6 weeks<br><br>**Resistance exercise:** **Volume:** Total duration (No info.); Stimuli duration (No info.) 3sess/week; 6 weeks<br><br>**Minimal education:** **Volume:** No info. of the duration	Pain intensity (VAS and WOMAC-Pain)	Aerobic group showed greater reduction than the no treatment group in VAS and WOMAC-Pain	Vig-AE < No treatment
					No differences found between aerobic and resistance group in VAS and WOMAC-Pain	Vig-AE ≈ Resistance
				Walking performance (6MWT)	No differences found between aerobic and no treatment groups	Vig-AE ≈ No Treatment
					No differences found between aerobic and resistance groups	Vig-AE ≈ Resistance
				Function in sit-to-stand (30s chair-to-stand)	No differences found between aerobic and no treatment groups	Vig-AE ≈ No Treatment
					No differences found between aerobic and resistance groups	Vig-AE ≈ Resistance
				Stiffness (WOMAC-Stiffness)	No differences found between aerobic and no treatment groups	Vig-AE ≈ No Treatment
					No differences found between aerobic and resistance groups	Vig-AE ≈ Resistance
				Disability associated with KOA (WOMAC-Total)	Greater reduction in disability in aerobic compared to no treatment group	Vig-AE < No Treatment
					No differences found between aerobic and resistance groups	Vig-AE ≈ Resistance
**Watanabe & Someya 2013 [40]**	**KOA diagnostic criteria:**	**Very light-to-light AE** (n_{allo}=15, n_{an}=12):	**Full-body-weight continuous walking:** **Condit. Intensity:** No	Pain intensity (VAS)	Greater reduction in body-weight-supported AE	Very light-light AE Full body weight > Very

Parallel RCT	Clinical (JOA score) and radiological (YCCS) **Presence of pain:** Probable, as it was considered as a component of clinical criteria	Age: 75 ± 7.6; F (n= 10); BMI: 22.7 ±3.1; Diabetes n/a; Hypertension: n/a **Very light-to-light AE** (n_{allo}=15, n_{an}=13): Age: 80 ± 5.9; F (n= 11); BMI: 22.2 ± 3.7; Diabetes n/a; Hypertension: n/a	**Final intensity:** Very light-to-light; 40-60% HR_{max} and 12-14 Borg (employed both parameters) **Monit. Device:** Poral RS100 **Volume:** Total duration (28 min); Stimuli duration (20 min); 2 sess/week for 6 weeks		compared to full body weight group	light-light Partial weight
				Walking performance (6MWT (m) and 10 m WT (s))	Greater distance in 6MWT and greater speed in 10m WT in the body-weight-supported group compared to full weight group	Very light-light Partial weight > Very light-light Full body weight
			**Body-weight-supported continuous walking:** **Condit. Intensity:** No **Final intensity:** Very light-to-light ;40-60% HR_{max} or 12-14 Borg employed both parameters) **Monit. Device:** Poral RS100 **Volume:** Total duration (28 min); Stimuli duration (20 min); 2 sess/week for 6 weeks	Function in sit-to-stand and walking (TUG)	No differences found between full weight and body-weight-supported groups	Very light-light Full body weight ≈ Very light-light Partial weight

Table S4. Data availability, extraction and estimations for meta-analyses.

Outcome measure	Analyses outcome measure of interest (k)	Original studies (k)	Comparisons eligible for meta-analysis (k)	Text/Table or Plot (k)	Included in the meta-analysis<br>Extractable (k)	 Raw extraction as Mean and SD (k)	 Instruments employed in NMA (k)
**Pain intensity**	Yes (12): (Arrieiro et al., 2019; Beckwée et al., 2015; Casilda-López et al., 2017; de Almeida et al., 2019, 2020; Ettinger et al., 1997; Lim et al., 2010; Messier et al., 1997; Øiestad et al., 2023; Salacinski et al., 2012; Samut et al., 2015; Watanabe & Someya, 2013) [26,28,29,30,32,34,36–40]	Yes (10): (Arrieiro et al., 2019; Beckwée et al., 2015; Casilda-López et al., 2017; de Almeida et al., 2019; Ettinger et al., 1997; Lim et al., 2010; Øiestad et al., 2023; Salacinski et al., 2012; Samut et al., 2015; Watanabe & Someya, 2013).[26,28,29,30,32,34,37–40]	Yes (7): (Beckwée et al., 2015; de Almeida et al., 2019; Ettinger et al., 1997; Lim et al., 2010; Øiestad et al., 2023; Salacinski et al., 2012; Samut et al., 2015). [28,30,32,34,37–39]	Text/Table (7): (Beckwée et al., 2015; de Almeida et al., 2019; Ettinger et al., 1997; Lim et al., 2010; Øiestad et al., 2023; Salacinski et al., 2012; Samut et al., 2015) [28,30,32,34,37–39].	Yes (7): (Beckwée et al., 2015; de Almeida et al., 2019; Ettinger et al., 1997; Lim et al., 2010; Øiestad et al., 2023; Salacinski et al., 2012; Samut et al., 2015) [28,30,32,34,37–39].	Yes (5): (de Almeida et al., 2019; Lim et al., 2010; Øiestad et al., 2023; Salacinski et al., 2012; Samut et al., 2015). [30,34,37–39] No (2): Mean estimated from median* (Beckwée et al., 2015) [28]; SD estimated from IQR** (Ettinger et al., 1997) [32].	VAS (2): (de Almeida et al., 2019; Samut et al., 2015) [30,39] VAS after 6 min normal walking (1): (Salacinski et al., 2012) [38]<br>NRS maximal the previous 24h (1): (Beckwée et al., 2015) [28] NRS average the previous 2 weeks (1): (Øiestad et al., 2023) [37]<br>BPI (1): (Lim et al., 2010). [34]<br>1-6 scale (1): (Ettinger et al., 1997). [32]
			No (3): Exclusive comparisons of groups with equal aerobic exercise intensities (Arrieiro et al., 2019; Casilda-López et al., 2017; Watanabe & Someya, 2013). [26,29,40]				
		No (2): Subsample of Ettinger et al., 2019 with fewer subjects (Messier et al., 1997) [32,36]; Same sample as de Almeida et al., 2019 (de Almeida et al., 2020)* [30,31]					
	No (3): (Bavardi Moghadam & Shojaedin, 2017; Keogh et al., 2018; Mangione et al., 1999). [27,33]						
**Walking performance**	Yes (11): (Arrieiro et al., 2019; Bavardi Moghadam & Shojaedin, 2017; Casilda-López et al., 2017; de Almeida et al., 2020; Ettinger et al., 1997; Keogh et al., 2018; Mangione et al., 1999; Messier	Yes (10): (Arrieiro et al., 2019; Bavardi Moghadam & Shojaedin, 2017; Casilda-López et al., 2017; de Almeida et al., 2020; Ettinger et al., 1997; Keogh et al., 2018; Mangione et al., 1999;	Yes (7): (Bavardi Moghadam & Shojaedin, 2017; de Almeida et al., 2020; Ettinger et al., 1997; Keogh et al., 2018; Mangione et al., 1999; Salacinski et al., 2012; Samut et al., 2015). [27,31–33,38,39]	Text/Table (7): (Bavardi Moghadam & Shojaedin, 2017; de Almeida et al., 2020; Ettinger et al., 1997;	Yes (7): (Bavardi Moghadam & Shojaedin, 2017; de Almeida et al., 2020; Ettinger et al., 1997; Keogh et	Yes (6): (Bavardi Moghadam & Shojaedin, 2017; de Almeida et al., 2020; Keogh et al., 2018; Mangione et al., 1999;	6MWT (4) (m): (Bavardi Moghadam & Shojaedin, 2017; Ettinger et al., 1997; Mangione et al., 1999; Samut et al., 2015). [27,32,39]<br>Normal walk speed in 3.66m

	et al., 1997; Salacinski et al., 2012; Samut et al., 2015; Watanabe & Someya, 2013). [26,27,29,31–33,36,38–40]	Salacinski et al., 2012; Samut et al., 2015; Watanabe & Someya, 2013). [26,27,29,31–33,38–40]		Keogh et al., 2018; Mangione et al., 1999; Salacinski et al., 2012; Samut et al., 2015). [27,31–33,38,39]	al., 2018; Mangione et al., 1999; Salacinski et al., 2012; Samut et al., 2015) [27,31–33,38,39].	Salacinski et al., 2012; Samut et al., 2015). [27,34,33,38,39]<br>No (1): SD estimated from SE‡ (Ettinger et al., 1997) [32]	(2) (m/s): (Keogh et al., 2018; Salacinski et al., 2012). [33,38]<br>40m walk test (1) (m/s): (de Almeida et al., 2020). [31]
			No (3): Exclusive comparisons of groups with equal aerobic exercise intensities (Arrieiro et al., 2019; Casilda-López et al., 2017; Watanabe & Someya, 2013). [26,29,40]				
		No (1): Subsample of Ettinger et al., 2019 with fewer subjects (Messier et al., 1997) [32,36]					
	No (4): (Beckwée et al., 2015; de Almeida et al., 2019; Lim et al., 2010; Øiestad et al., 2023). [28,30,34,37]						

Methodological Quality Assessment with PEDro scale

Studies	Item nº2	Item nº3	Item nº4	Item nº5	Item nº6	Item nº7	Item nº8	Item nº9	Item nº10	Item nº11	Total	Kappa
Arriero 2019	Yes	Yes	Yes	No	No	Yes	Yes	Yes	Yes	Yes	8	0.737
Bavardi Moghadam & Shojaedin 2017	Yes	No	Yes	No	No	Yes	Yes	No	Yes	Yes	5	0.8
Beckwee 2015	Yes	Yes	Yes	No	No	No	No	Yes	No	Yes	5	0.8
Casilda−López 2017	Yes	Yes	Yes	No	No	Yes	Yes	Yes	Yes	Yes	8	1
De Almeida 2019	Yes	Yes	Yes	No	No	Yes	Yes	No	Yes	Yes	7	0.737
De Almeida 2020	Yes	No	Yes	No	No	Yes	Yes	No	Yes	Yes	6	0.545
Ettinger 1997	Yes	Yes	Yes	No	No	No	No	Yes	Yes	Yes	7	0.583
Keogh 2018	Yes	Yes	Yes	No	No	No	No	No	Yes	Yes	5	1
Lim 2010	Yes	Yes	Yes	No	No	Yes	Yes	Yes	Yes	Yes	8	1
Mangione 1999	Yes	No	Yes	No	No	No	No	No	Yes	Yes	4	0.6
Messier 1997	Yes	No	Yes	No	No	No	No	No	Yes	Yes	4	0.8
Øiestad 2023	Yes	Yes	Yes	No	No	No	No	Yes	Yes	Yes	7	0.737
Salacinski 2012	Yes	Yes	Yes	No	No	No	No	Yes	Yes	Yes	6	1
Samut 2015	Yes	No	Yes	No	No	Yes	Yes	No	No	Yes	4	0.615
Watanabe & Someya 2013	Yes	Yes	Yes	No	No	No	No	No	No	Yes	5	0.8

Figure S1. Methodological quality assessment with PEDro scale [26–40].

Pain Intensity

Study	D1	D2	D3	D4	D5	Total	Kappa
Watanabe & Someya 2013	✓	✗	⚠	⚠	✗	✗	0.29
Samut 2015	⚠	✗	✓	⚠	✗	✗	0.64
Salacinski 2012	✓	✗	✓	⚠	⚠	✗	0.57
Øiestad 2023	✓	✓	✓	⚠	✓	⚠	0.06
Messier 1997	⚠	✗	✗	⚠	✗	✗	0.12
Lim 2010	✓	⚠	✓	⚠	✗	✗	0.75
Ettinger 1997	✓	⚠	⚠	⚠	✗	✗	0.8
De Almeida 2019	✓	✓	✓	⚠	⚠	⚠	0.71
Casilda−López 2017	✓	✓	✓	⚠	⚠	⚠	0.78
Beckwee 2015	✓	⚠	✓	⚠	✗	✗	0.58
Arrieiro 2019	✓	✓	✓	⚠	✗	✗	0.78

Walking performance

Study	D1	D2	D3	D4	D5	Total	Kappa
Arrieiro 2019	✓	✓	✓	⚠	✗	✗	0.76
Bavardi Moghadam 2017	⚠	⚠	✗	⚠	✗	✗	0.44
Casilda−López 2017	✓	✓	✓	⚠	⚠	⚠	0.76
De Almeida 2020	⚠	⚠	⚠	⚠	⚠	✗	0
Ettinger 1997	✓	⚠	⚠	⚠	✗	✗	1
Keogh 2018	✓	✗	⚠	⚠	⚠	✗	0.33
Mangione 1999	⚠	✗	✗	⚠	✗	✗	0.35
Messier 1997	⚠	✗	✗	⚠	✗	✗	0.12
Salacinski 2012	✓	✗	✓	⚠	⚠	✗	0.57
Samut 2015	⚠	✗	✓	⚠	✗	✗	0.64
Watanabe & Someya 2013	✓	✗	⚠	⚠	✗	✗	0.4

Sit−to−Stand performance

Study	D1	D2	D3	D4	D5	Total	Kappa
De Almeida 2020	⚠	⚠	⚠	⚠	⚠	✗	0
Keogh 2018	✓	✗	⚠	⚠	⚠	✗	0.33
Mangione 1999	⚠	✗	✗	⚠	✗	✗	0.35
Samut 2015	⚠	✗	✓	⚠	✗	✗	0.64

Linear walk with Sit−to−Stand performance

Study	D1	D2	D3	D4	D5	Total	Kappa
Bavardi Moghadam 2017	⚠	⚠	✗	⚠	✗	✗	0.44
Keogh 2018	✓	✗	⚠	⚠	⚠	✗	0.33
Watanabe & Someya 2013	✓	✗	⚠	⚠	✗	✗	0.4

Stiffness

Study	D1	D2	D3	D4	D5	Total	Kappa
Arrieiro 2019	✓	✓	✓	⚠	✗	✗	0.78
Casilda−López 2017	✓	✓	✓	⚠	⚠	⚠	0.78
De Almeida 2020	⚠	⚠	⚠	⚠	⚠	✗	0.24
Salacinski 2012	✓	✗	✓	⚠	⚠	✗	0.57
Samut 2015	⚠	✗	✓	⚠	✗	✗	0.64

Disability

Study	D1	D2	D3	D4	D5	Total	Kappa
Casilda−López 2017	✓	✓	✓	⚠	⚠	⚠	0.78
De Almeida 2020	⚠	⚠	⚠	⚠	⚠	✗	0.24
Keogh 2018	✓	✗	⚠	⚠	⚠	✗	0.33
Lim 2010	✓	⚠	✓	⚠	✗	✗	0.75
Salacinski 2012	✓	✗	✓	⚠	⚠	✗	0.57
Samut 2015	⚠	✗	✓	⚠	✗	✗	0.64

Figure S2. Risk of bias assessment with ROB 2.0 tool for every outcome of interest [26–40].

✓, low risk of bias judgment; ⚠, some concerns judgment; ✗, high risk of bias judgment. D1: domain 1, risk of bias arising from the randomization process; D2: domain 2, risk of bias due to deviations from the intended interventions (effect of assignment to intervention); D3: domain 3, risk of bias due to missing outcome data; D4: domain 4, risk of bias in measurement of the outcome; D5: domain 5, risk of bias in selection of the reported result.

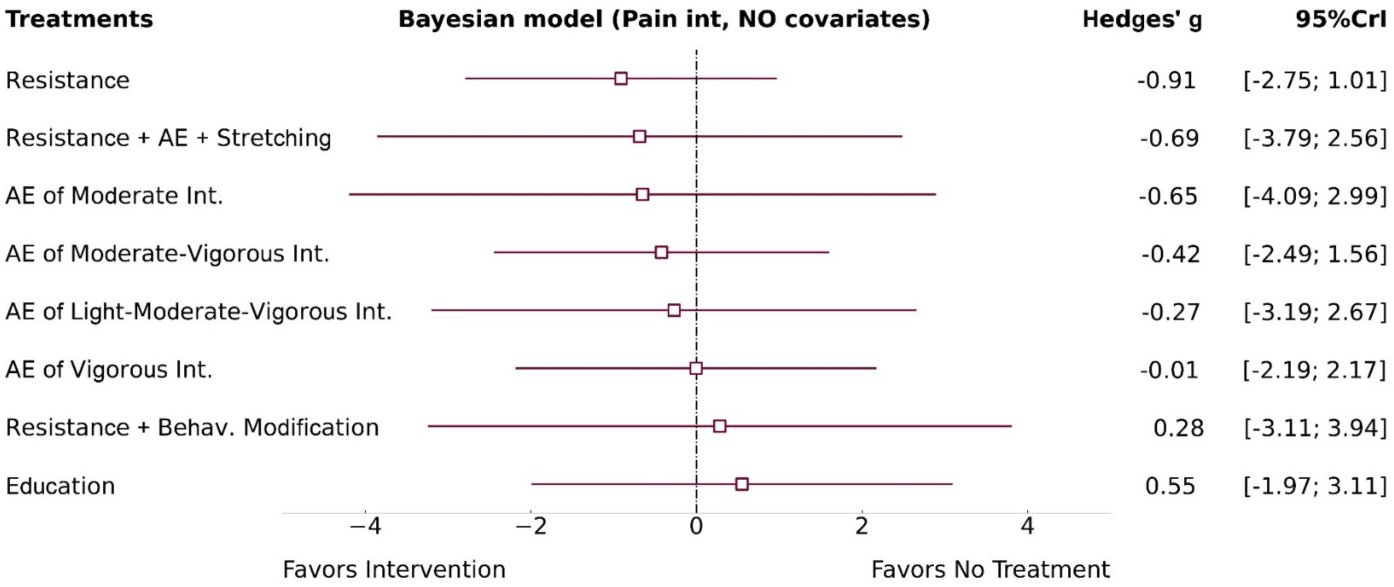

Figure S3. Forest plot of NMA Model 1 (NMA without covariates) for pain intensity

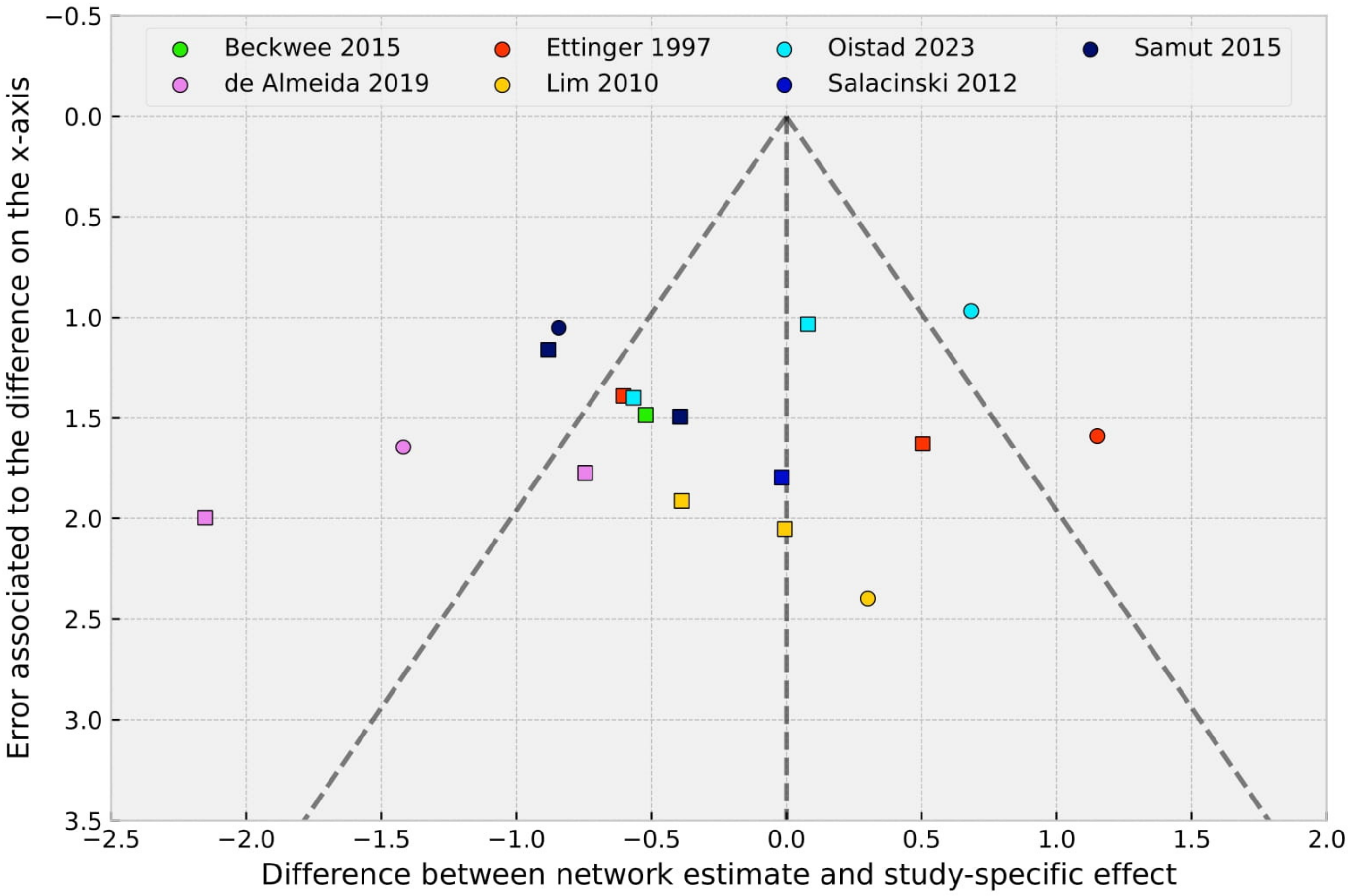

Figure S4. Funnel plot of NMA Model 1 (NMA without covariates) for pain intensity [28,30,32,34,37–39].

Triangles represent comparisons between two aerobic exercise interventions. Circles indicate comparisons between two non-aerobic exercise interventions. Squares represent comparisons between an aerobic exercise intervention and a non-aerobic exercise intervention.

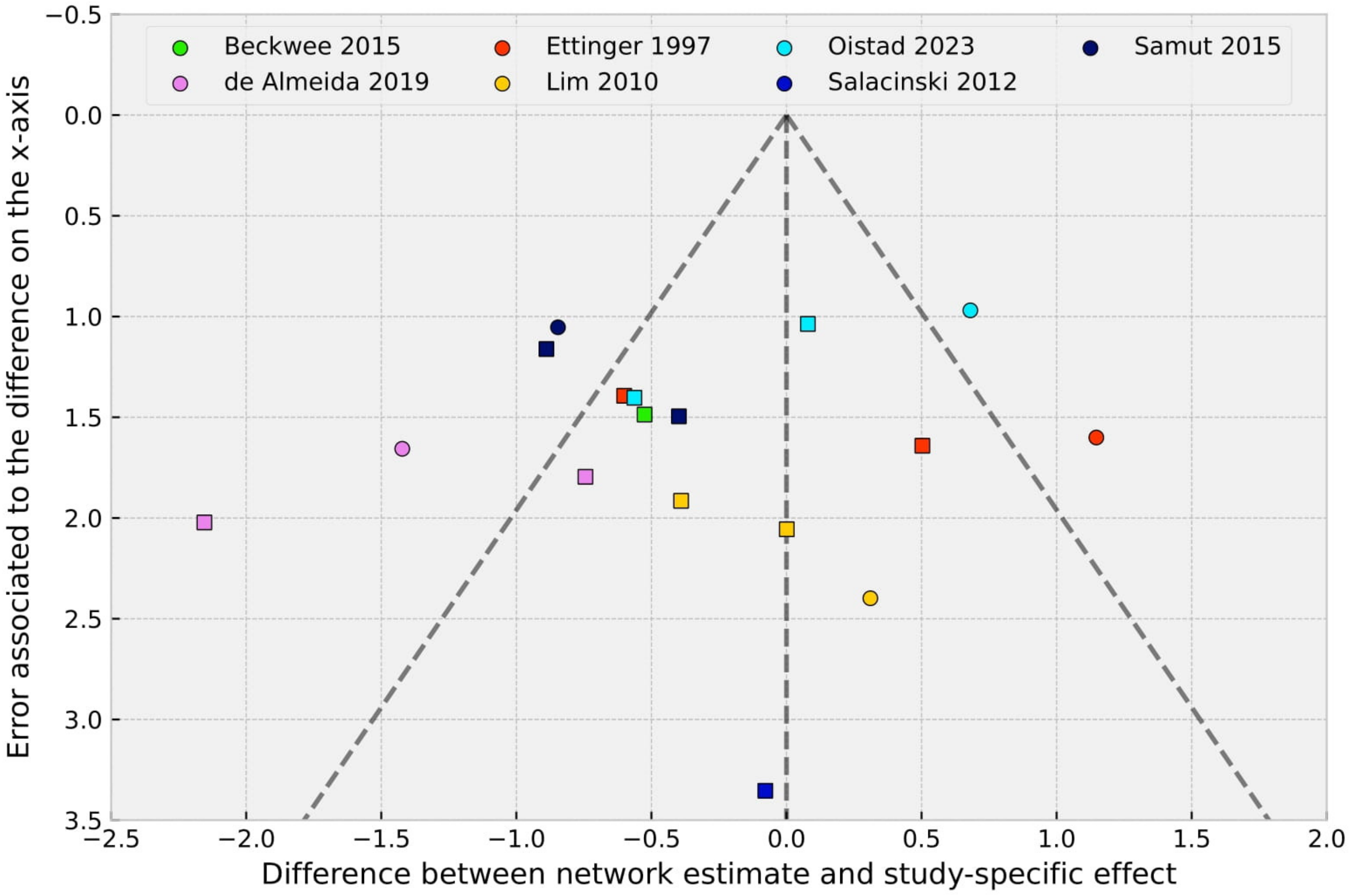

Figure S5. Funnel plot of NMA Model 2 (NMA with covariates) for pain intensity [28,30,32,34,37–39].

Triangles represent comparisons between two aerobic exercise interventions. Circles indicate comparisons between two non-aerobic exercise interventions. Squares represent comparisons between an aerobic exercise intervention and a non-aerobic exercise intervention.

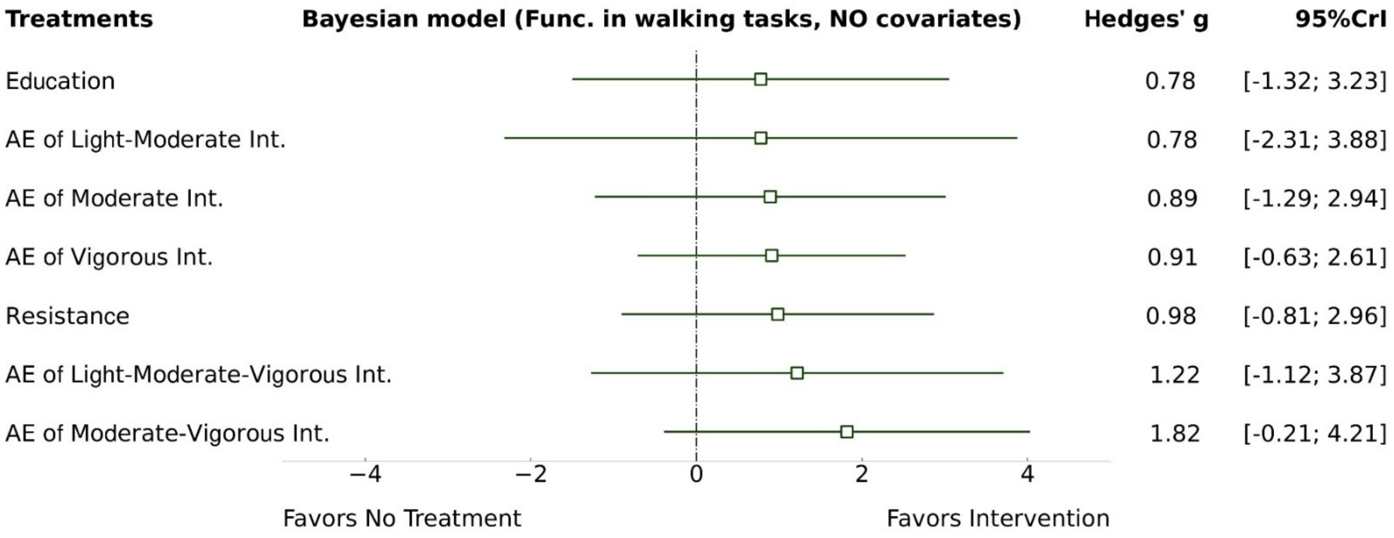

Figure S6. Forest plot of NMA Model 1 (NMA without covariates) for walking performance

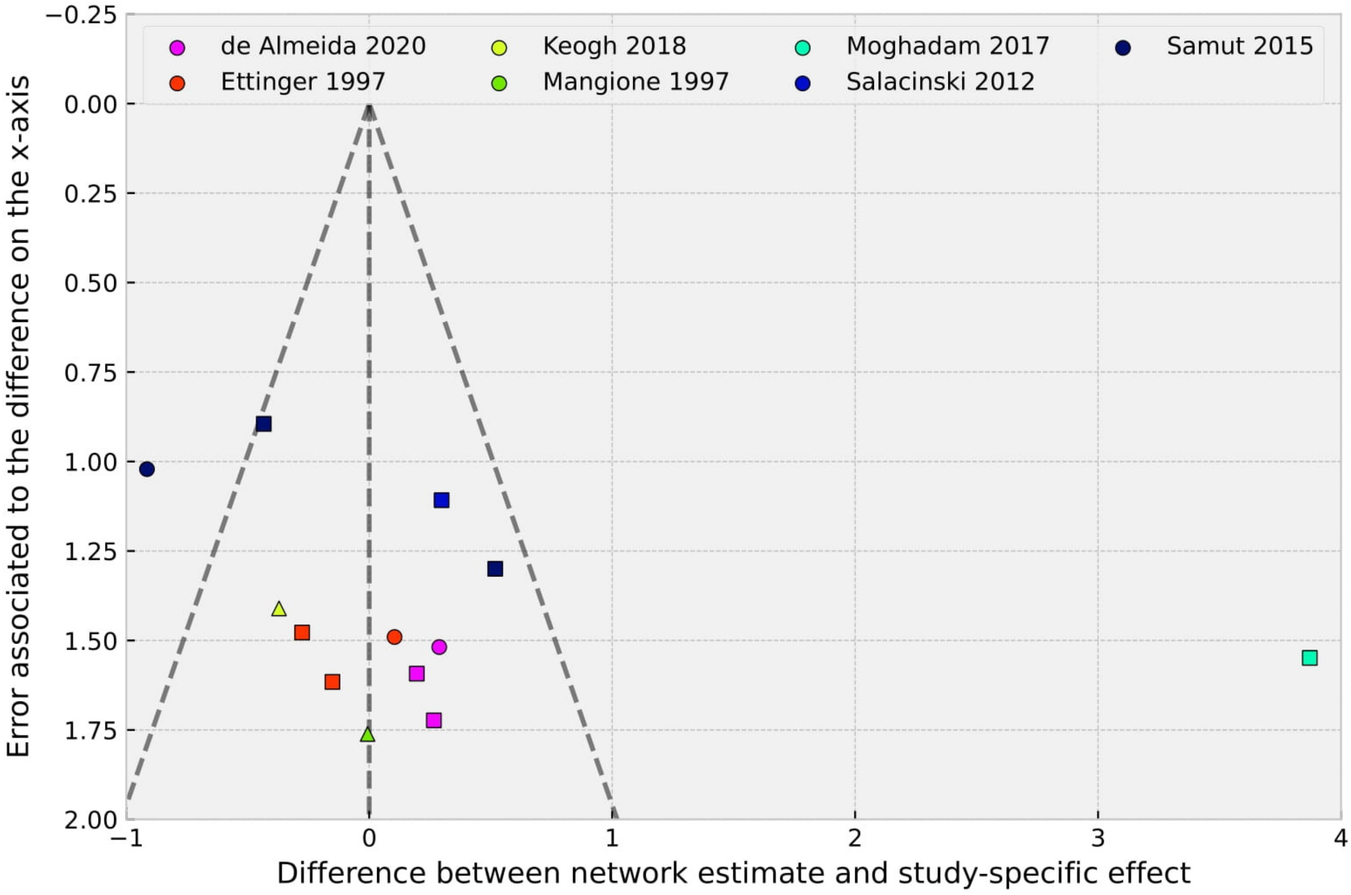

Figure S7. Funnel plot of NMA Model 1 (NMA without covariates) for walking performance [27,31–33,35,38,39].

Triangles represent comparisons between two aerobic exercise interventions. Circles indicate comparisons between two non-aerobic exercise interventions. Squares represent comparisons between an aerobic exercise intervention and a non-aerobic exercise intervention.

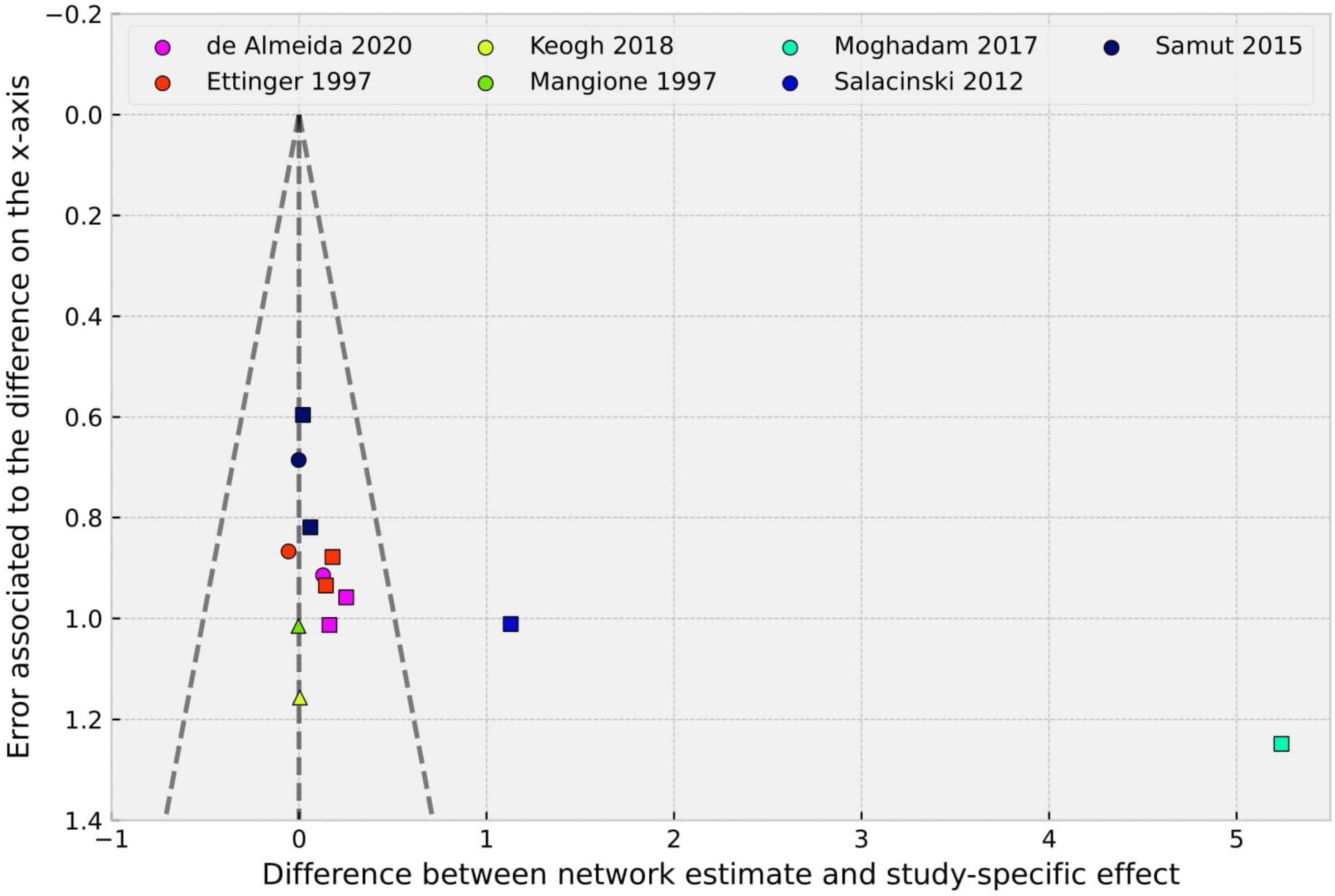

Figure S8. Funnel plot of NMA Model 2 (NMA with covariates) for walking performance [27,31–33,35,38,39]

Triangles represent comparisons between two aerobic exercise interventions. Circles indicate comparisons between two non-aerobic exercise interventions. Squares represent comparisons between an aerobic exercise intervention and a non-aerobic exercise intervention.

Supplementary Material Text 1. Description of NMA methodology.

The primary analysis modeled different AE intensities as nodes in the network. Additionally, based on available data from the original studies, a second NMA was conducted incorporating the covariates weekly frequency (w_f), and number of weeks (w_n). The relative efficacy, d_{bi}, of treatment *i* was modeled as:

$$d_{bi} = d_{bi}^{0} + c_f w_f + c_n w_n$$

where transitivity holds for baseline estimates $d_{ij}^{0} = d_{bj} - d_{bi}$, and coefficients c_f and c_w are inferred from the model. For non-aerobic treatments, both w_f and w_n were set to zero.

Trial-specific efficacy $\Delta_{t,ij}$ was sampled from a normal distribution $N(d_{ij}, \tau^2)$, where τ is the heterogeneity parameter. Patient-specific efficacy was then sampled from $N(d_{ij}, \tau^2)$ where $\sigma_{t,ij}^2$ is the trial-specific variance extracted from the data. These two levels of randomness make the model a random effects model.

The prior distribution for each d_{bi} was a normal distribution centered at zero with variance of 100. For τ, we used a uniform distribution over [0, 5]. Both priors were chosen to be non-informative.

For both the forest plots and the pairwise comparisons, results were expressed as Hedges' g, with the corresponding 95% credible intervals (CrI), p-values, and τ estimates. Direct estimates were obtained via a weighted average of all available direct comparisons, where the weights were the inverse of the corresponding standard errors.

To evaluate the potential for publication bias and inconsistency within the network, we generated comparison-adjusted funnel plots for each outcome. For each pairwise comparison between treatments within a study, we calculated the difference between the study's observed effect size and the corresponding effect estimate from the NMA model. The standard error of this difference was plotted on the y-axis. In the absence of significant bias or inconsistency, the data points are expected to lie within the 95% CrI of the funnel, which is bounded by the lines x = ± 1.96 * SE. Points located outside this funnel indicate a greater-than-expected discrepancy between the direct and network estimates, which can be indicative of publication bias, heterogeneity, or other systematic sources of inconsistency. The data points are colored by study and shaped to denote the type of intervention comparison: triangles for comparisons between two AE interventions, circles for comparisons between two non-AE interventions, and squares for mixed comparisons between an AE and a non-AE intervention.

Supplementary Material Text 2. Computation of Hedges' g and its exact variance.

The standardized mean difference was computed using Hedges' unbiased estimator $g_{t,ij}$, which adjusts Cohen's $d_{t,ij}$ for small sample bias. First, Cohen's d was calculated as the difference between experimental and control group means divided by the pooled standard deviation (Equation 3 in Hedges, 1982):

$$d_{t,ij} = \frac{x_{t,i} - x_{t,j}}{\sigma^*_{t,ij}}$$

With the pooled standard deviation defined as:

$$\sigma^*_{t,ij} = \sqrt{\frac{(N_{t,i} - 1) \cdot \sigma^2_{t,ij} + (N_{t,j} - 1) \cdot \sigma^2_{t,j}}{N_{t,i} + N_{t,j} - 2}}$$

To correct for bias in small samples, $d_{t,ij}$ was multiplied by the correction factor $c(m)_{t,ij}$, where $m_{t,ij} = N_{t,i} + N_{t,j} - 2$. Following in Equation 5:

$$c(m)_{t,ij} \approx 1 - \frac{3}{4m_{t,ij} - 1}$$

This yielded the Hedges' g $(g_{t,ij})$ corrected estimator:

$$\Delta_{t,ij} = c(m)_{t,ij} \cdot d_{t,ij}$$

The exact variance of $g_{t,ij}$ was estimated using the formula provided in Equation 6 of Hedges (1982):

$$\mathrm{Var}(g_{t,ij}) = \frac{\left[c(m)_{t,ij}\right]^2 \cdot m_{t,ij} \cdot (1 + \tilde{n}_{t,ij} \cdot g^2_{t,ij})}{(N_{t,i} + N_{t,j} - 4) \cdot \tilde{n}_{t,ij}} - g^2_{t,ij}$$

with $\tilde{n}_{t,ij}$ defined as:

$$\tilde{n}_{t,ij} = \frac{N_{t,i} \cdot N_{t,j}}{N_{t,i} + N_{t,j}}$$

Supplementary Material Text 3. Python syntax for computing Bayesian NMA, pairwise comparisons and funnel plots.

```
import numpy as np
import pandas as pd
import matplotlib.pyplot as plt
from matplotlib import cm, colormaps
import os
import pickle
import warnings
from tqdm import tqdm
import pickle
import time
from multiprocessing import cpu_count
import networkx as nx
import pymc3 as pm
import arviz as az


# Auxiliary function to compute Hedge's G
# the inputs are the efficacies, the errors, and the samples sizes respectively
def hedgesG(eff1, eff2, sd1, sd2, N1, N2):
    m = N1 + N2 - 2
    pool_sd = np.sqrt(((N1 - 1) * sd1**2 + (N2 - 1) * sd2**2) / m)
    c = 1 - (3 / (4 * m - 1))
    g12 = c * (eff2 - eff1) / pool_sd
    err12 = np.sqrt(((N1 + N2) / (N1 * N2)) + (0.5 * g12**2 / (N1 + N2)))
    return g12, err12


# Main class of the module, actually running the Bayesian NMA
class koaBayesianNMA():

    # init function
    def __init__(self, edgelist, edgelist_map=None):
        # the edgelist format is the same suggested by its map:
        # [study, treatment A, treatment B, trial-specific relative efficacy, its error,
        #  weekly frequency of A, weekly frequency of B, n of weeks of A, n of weeks of B]
        # The covariates (freq and weeks) are set to zero in treatments where they are absent
        if edgelist_map == None:
            self.edgelist_map = {'study': 'study', 'T_a': 'T_a', 'T_b': 'T_b',
                                 'delta_ab': 'delta_ab', 'std_ab': 'std_ab',
                                 'freq_a': 'freq_a', 'freq_b': 'freq_b',
                                 'weeks_a': 'weeks_a', 'weeks_b': 'weeks_b'}
        elif any(('study' not in edgelist_map.keys(), 'T_a' not in edgelist_map.keys(),
                  'T_b' not in edgelist_map.keys(), 'delta_ab' not in edgelist_map.keys(),
                  'std_ab' not in edgelist_map.keys(), 'freq_a' not in edgelist_map.keys(),
                  'freq_b' not in edgelist_map.keys(), 'weeks_a' not in edgelist_map.keys(),
                  'weeks_b' not in edgelist_map.keys())):
            raise Exception('Ill-defined edgelist_map!')
        else:
            self.edgelist_map = edgelist_map
        self.edgelist = edgelist
        self.treatments = np.unique(np.concatenate((edgelist[self.edgelist_map['T_a']],
                                                    edgelist[self.edgelist_map['T_b']])))
```

```
        self.baseline = self.treatments[0]
        self.n_d = self.treatments.shape[0] - 1
        self.d_to_idx = dict(zip(self.treatments[1:], range(self.n_d)))
        self.idx_to_d = dict(zip(range(self.n_d), self.treatments[1:]))
        self.n_trials = self.edgelist.shape[0]
        self.n_studies = np.unique(self.edgelist[self.edgelist_map['study']]).shape[0]
        self.delta_shapes = []
        self.X_matlist = []
        self.delta_matlist = []
        self.std_matlist = []
        self.f_matlist, self.w_matlist = [], []
        for study, ssdf in edgelist.groupby(self.edgelist_map['study']):
            if ssdf.shape[0] > 1:
                nodes = np.unique(np.concatenate((ssdf[self.edgelist_map['T_a']],
                                                  ssdf[self.edgelist_map['T_b']])))
                baseline = nodes[0]
            else:
                baseline = None
            deltas, stds, sub_X, sub_f, sub_w = [], [], [], [], []
            for _, line in ssdf.iterrows():
                fline, wline = np.zeros(self.n_d), np.zeros(self.n_d)
                if baseline is None:
                    baseline = np.unique((line[self.edgelist_map['T_a']],
                                          line[self.edgelist_map['T_b']]))[0]
                xline = np.zeros(self.n_d)
                if baseline != self.baseline:
                    xline[self.d_to_idx[baseline]] = -1
                if line[self.edgelist_map['T_a']] != baseline:
                    xline[self.d_to_idx[line[self.edgelist_map['T_a']]]] = 1
                    f = line[self.edgelist_map['freq_a']]
                    fline[self.d_to_idx[line[self.edgelist_map['T_a']]]] = f
                    w = line[self.edgelist_map['weeks_a']]
                    wline[self.d_to_idx[line[self.edgelist_map['T_a']]]] = w
                    deltas.append(-line[self.edgelist_map['delta_ab']])
                else:
                    xline[self.d_to_idx[line[self.edgelist_map['T_b']]]] = 1
                    f = line[self.edgelist_map['freq_b']]
                    fline[self.d_to_idx[line[self.edgelist_map['T_b']]]] = f
                    w = line[self.edgelist_map['weeks_b']]
                    wline[self.d_to_idx[line[self.edgelist_map['T_b']]]] = w
                    deltas.append(line[self.edgelist_map['delta_ab']])
                sub_X.append(xline)
                stds.append(line[self.edgelist_map['std_ab']])
            self.delta_shapes.append(len(deltas))
            self.delta_matlist.append(np.array(deltas))
            self.std_matlist.append(np.array(stds))
            self.X_matlist.append(np.array(sub_X))
            fline = np.nan_to_num(fline)
            wline = np.nan_to_num(wline)
            self.f_matlist.append(fline)
            self.w_matlist.append(wline)
        self.model = None

    # builds the NMA model
    def modelBuilder(self, noninfo_sigma=1e4, tau_max=5, simple=False, student_t=False):
        # noninfo_sigma is the sigma of the prior
        # tau_max is the upper limit of the tau flat prior [0, tau_max]
```

```
    # simple=True tells the function to include the covariates in the inference
    # student-t can be used to replace the Gaussians
    self.model = pm.Model()
    with self.model:
      d_0 = pm.Normal("d_0", mu=np.zeros(self.n_d), shape=self.n_d, sigma=noninfo_sigma)
      if simple:
        c_f = np.zeros(self.n_d)
        c_w = np.zeros(self.n_d)
      else:
        c_f = np.ones(self.n_d) * pm.Normal("c_f", mu=0, sigma=noninfo_sigma)
        c_w = np.ones(self.n_d) * pm.Normal("c_w", mu=0, sigma=noninfo_sigma)
      tau = pm.Uniform("tau", lower=0, upper=tau_max)
      if student_t:
        nu = pm.Uniform(f"nu", lower=0, upper=50)
      for i in tqdm(range(len(self.X_matlist)), colour='green'):
        xish = self.X_matlist[i].shape[0]
        d = d_0 + pm.math.dot(c_w,self.w_matlist[i]) + pm.math.dot(c_f,self.f_matlist[i])
        if xish == 1:
          if student_t:
            deltai = pm.StudentT(f"deltas_{i}", sigma=tau + self.std_matlist[i][0],
                          nu=nu, mu=pm.math.dot(self.X_matlist[i][0], d),
                          observed=self.delta_matlist[i][0])
          else:
            deltai = pm.Normal(f"deltas_{i}", sigma=tau + self.std_matlist[i][0],
                         mu=pm.math.dot(self.X_matlist[i][0], d),
                         observed=self.delta_matlist[i][0])
        else:
          sigmat = (np.ones((xish, xish)) + np.identity(xish)) * 0.5 * (tau ** 2)
          varmat = np.diag(self.std_matlist[i]**2)
          if student_t:
            deltai = pm.MvStudentT(f"deltas_{i}", Sigma=sigmat+varmat, shape=xish,
                           nu=nu, observed=self.delta_matlist[i],
                           mu=pm.math.matrix_dot(self.X_matlist[i], d))
          else:
            deltai = pm.MvNormal(f"deltas_{i}", cov=sigmat+varmat, shape=xish,
                          mu=pm.math.matrix_dot(self.X_matlist[i], d),
                          observed=self.delta_matlist[i])

  # actually runs the model
  def runBayes(self, niters=2500, burn_in=1000, chains=4, save_path=None, simple=False,
         noninfo_sigma=1e4, tau_max=5, student_t=False, cores=None):
    # niters is the number of MCMC samples
    # burn_in is the number of tuning samples
    # chains is the number of MC chains
    # if save_path is not None, the trace is saved there
    # cores is the number of cores used by the model
    # all the other variables are the same as modelBuilder
    startime = time.time()
    self.modelBuilder(noninfo_sigma=noninfo_sigma, tau_max=tau_max,
              simple=simple, student_t=student_t)
    print('Starting to sample (this will take a while, especially after the prog-bar)...')
    with self.model:
      trace = pm.sample(niters, tune=burn_in, cores=cores,
                chains=chains, return_inferencedata=True)
    print('Done!')
    elapstime = (time.time() - startime) // 60
    print(f"\nBayesian sampling is over! [tte = {elapstime} mins]")
```

```
        if save_path is not None:
            pickle.dump(trace, open(save_path, 'wb'))
        return trace


# extracts the result table from the trace
def resulTable(trace, d_names, save_path=None, show=True, simple=False, student_t=False):
    # trace output from the main class
    # d_names are the names of the treatments, in the same order as they were in the main class
    # if save_path is not None, the trace is saved there
    # if show=True it prints the table
    # simple and student-t should match the ones in the main class
    var_names = ['d_0', 'tau']
    if student_t:
        var_names.append('nu')
    if not simple:
        var_names.extend(['c_f', 'c_w'])
    df = az.summary(trace, var_names=var_names, hdi_prob=0.95)
    namecol = pd.Series(np.concatenate((d_names, var_names[1:])), name='variable')
    df = pd.concat((namecol, df.reset_index(drop=True)), axis=1)
    if save_path is not None:
        df.to_csv(save_path, index=None)
    if show:
        display(df)
    return df


# generates the forest plot
def forestPlotter(df, n_d, title='', d_color='xkcd:scarlet', save_path=None,
                  xlim=5, fsize=25, good_effect=True):
    # df is the result table from the previous function
    # n_d is the number of treatments
    # title will be printed above the plot in the figure
    # d_color is the color of the treatmetn bars
    # if save_path is not None, the trace is saved there
    # xlim is the limit of the x-axis (treatment efficacy)
    # fsize is the fontsize
    # if good_effect=True a positive value of d favors intervention
    # if good_effect=False a positive value of d favors "No Treatment"
    figa, ax = plt.subplots(1, 1, figsize=(16, n_d + 1))
    hdi = 0.5 * (df['hdi_97.5%'].iloc[:n_d] - df['hdi_2.5%'].iloc[:n_d])
    dy = [*reversed(range(df.shape[0] - n_d, df.shape[0]))]
    xtreats = df['mean'].iloc[:n_d]
    idx = np.argsort(xtreats)
    ax.errorbar(xtreats[idx], dy, xerr=hdi[idx], color=d_color, linewidth=2,
                zorder=5, linestyle='')
    ax.scatter(xtreats[idx], dy, marker='s', s=150, facecolor='white',
               edgecolor=d_color, zorder=50, linewidth=2)
    renames = {'EP': 'ED', 'LMV': 'AE_LIGHT_MOD_VIG', 'LM': 'AE_LIGHT_MOD', 'M': 'AE_MOD',
               'MV': 'AE_MOD_VIG', 'NT': 'NT', 'RE': 'RE', 'V': 'AE_VIG',
               'REBLM': 'RE_BEHAV', 'REASE': 'RE_AE_STRETCH'}
    for i, di in zip(idx, dy):
        ax.text(-xlim*1.67, di, renames[df['variable'].iloc[i]],
                ha='left', va='center', fontsize=fsize)
        ax.text(xlim*1.2, di, np.round(df['mean'].iloc[i], 2),
                ha='right', va='center', fontsize=fsize)
        ax.text(xlim*1.67, di, f"[{df['hdi_2.5%'].iloc[i]:.2f}; {df['hdi_97.5%'].iloc[i]:.2f}]",
```

```
            ha='right', va='center', fontsize=fsize)
    ax.text(-xlim*1.67, dy[0]+1, 'Treatments', fontweight='bold',
            ha='left', va='center', fontsize=fsize)
    ax.text(0, dy[0]+1, title, fontweight='bold',
            ha='center', va='center', fontsize=fsize)
    ax.text(xlim*1.2, dy[0]+1, 'SMD', fontweight='bold',
            ha='right', va='center', fontsize=fsize)
    ax.text(xlim*1.67, dy[0]+1, '95%-CI', fontweight='bold',
            ha='right', va='center', fontsize=fsize)
    if good_effect:
        ax.text(-xlim, dy[-1]-1.5, 'Favors No Treatment', fontsize=fsize, ha='left', va='center')
        ax.text(xlim, dy[-1]-1.5, 'Favors Intervention', fontsize=fsize, ha='right', va='center')
    else:
        ax.text(-xlim, dy[-1]-1.5, 'Favors Intervention', fontsize=fsize, ha='left', va='center')
        ax.text(xlim, dy[-1]-1.5, 'Favors No Treatment', fontsize=fsize, ha='right', va='center')
    ax.axvline(0, color='black', linewidth=1.5, zorder=10, linestyle='-.')
    ax.set_yticks(dy)
    ax.set_yticklabels('', fontsize=17)
    ax.tick_params(axis='x', labelsize=fsize)
    ax.set_ylim(dy[-1] - 0.5, dy[0] + 0.5)
    ax.set_xlim(-xlim, xlim)
    ax.set_facecolor('white')
    ax.yaxis.set_visible(False)
    ax.grid(False)
    for spine in ['top', 'left', 'right']:
        ax.spines[spine].set_visible(False)
    if save_path is not None:
        plt.savefig(save_path, bbox_inches='tight')
    plt.show()


# infers direct and indirect estimates
def directIndirectEstimator(edgelist, resultable, niters=10000, burn_in=20000, simple=True,
                            save_path=None):
    # edgelist should be in the same format as the main class
    # resultable is the output of the same-name function
    # niters is the number of MCMC samples
    # burn_in is the number of tuning samples
    # if save_path is not None, the trace is saved there
    nodelabels = {'Education protocol': 'EP', 'Light-to-Moderate-to-Vigorous': 'LMV',
                  'Light-to-Moderate': 'LM', 'Moderate': 'M', 'Moderate-to-Vigorous': 'MV',
                  '0_ No treatment': 'NT', 'Resistance exercise': 'RE', 'Vigorous': 'V',
                  'Resistance exercise and Behavioral-lifestyle modification': 'REBLM',
                  'Resistance, Aerobic, and Stretching exercises': 'REASE'}
    nodes = np.unique(np.concatenate((edgelist['T_a'], edgelist['T_b'])))
    N, M = len(nodes), edgelist.shape[0]
    d_nma = np.concatenate(([0], resultable['mean'].values[:N-1]))
    std_nma = np.concatenate(([0], resultable['sd'].values[:N-1]))
    # d5_nma = np.concatenate(([0], resultable['hdi_2.5%'].values[:N-1]))
    # d95_nma = np.concatenate(([0], resultable['hdi_97.5%'].values[:N-1]))
    if simple:
        tau_nma = resultable['mean'].values[-1]
        tausd_nma = resultable['mean'].values[-1]
    else:
        tau_nma = resultable['mean'].values[-3]
        tausd_nma = resultable['mean'].values[-3]
    G0 = nx.MultiGraph()
```

```
G0.add_nodes_from(nodes)
print(f"Nodes: {[nodelabels[n] for n in nodes]}\n")
final_df = []
final_cols = ['node_a', 'node_b', 'direct_est', 'direct_std', 'direct_2.5%',
              'direct_97.5%', 'indirect_est', 'indirect_std', 'indirect_2.5%',
              'indirect_97.5%', 'indirect_tau', 'indirect_tau_std', 'mixed_est',
              'mixed_std', 'mixed_2.5%', 'mixed_97.5%', 'mixed_tau', 'mixed_tau_std']
totals = N * (N - 1) // 2
c = 1
start = time.time()
for i in range(N):
    for j in range(i + 1, N):
        print(f"EVALUATING {nodelabels[nodes[i]]}-{nodelabels[nodes[j]]} ({c} / {totals})")
        G = G0.copy()
        ind_df = []
        dir_df = []
        for _, line in edgelist.iterrows():
            if line['T_a'] == nodes[i] and line['T_b'] == nodes[j]:
                dir_df.append(line.values)
            elif line['T_a'] == nodes[j] and line['T_b'] == nodes[i]:
                dir_df.append(line.values)
            else:
                G.add_edge(line['T_a'], line['T_b'])
                ind_df.append(line.values)
        if len(ind_df) == M:
            dir_d = float('nan')
            dir_std = float('nan')
            dir_5 = float('nan')
            dir_95 = float('nan')
            ind_d = d_nma[j] - d_nma[i]
            ind_std = np.sqrt(std_nma[j]**2 + std_nma[i]**2)
            ind_5 = ind_d - 1.96 * ind_std
            ind_95 = ind_d + 1.96 * ind_std
            ind_tau = tau_nma
            ind_tausd = tausd_nma
        elif not nx.is_connected(G):
            dir_df = pd.DataFrame(dir_df, columns=edgelist.columns)
            dir_d = np.average(dir_df['delta_ab'], weights=1/dir_df['std_ab'])
            dir_std = (np.sum(dir_df['std_ab']**-1))**-1
            dir_5 = dir_d - 1.96 * dir_std
            dir_95 = dir_d + 1.96 * dir_std
            ind_d = float('nan')
            ind_std = float('nan')
            ind_5 = float('nan')
            ind_95 = float('nan')
            ind_tau = float('nan')
            ind_tausd = float('nan')
        else:
            dir_df = pd.DataFrame(dir_df, columns=edgelist.columns)
            dir_d = np.average(dir_df['delta_ab'], weights=1/dir_df['std_ab'])
            dir_std = (np.sum(dir_df['std_ab']**-1))**-1
            dir_5 = dir_d - 1.96 * dir_std
            dir_95 = dir_d + 1.96 * dir_std
            ind_df = pd.DataFrame(ind_df, columns=edgelist.columns)
            ind_nma = UnivariateBayesianNMA(pain_df)
            trace = ind_nma.runBayes(niters=niters, burn_in=burn_in, chains=4, simple=simple,
                        student_t=False, noninfo_sigma=10, save_path=None)
```

```
            ind_table = resulTable(trace, nodes, show=False, simple=simple,
                                   student_t=False, save_path=None)
            d_vec = np.concatenate(([0], ind_table['mean'].values[:N-1]))
            std_vec = np.concatenate(([0], ind_table['sd'].values[:N-1]))
            ind_d = d_vec[j] - d_vec[i]
            ind_std = np.sqrt(std_vec[j]**2 + std_vec[i]**2)
            ind_5 = ind_d - 1.96 * ind_std
            ind_95 = ind_d + 1.96 * ind_std
            if simple:
                ind_tau = ind_table['mean'].values[-1]
                ind_tausd = ind_table['mean'].values[-1]
            else:
                ind_tau = ind_table['mean'].values[-3]
                ind_tausd = ind_table['mean'].values[-3]
        nma_d = d_nma[j] - d_nma[i]
        nma_std = np.sqrt(std_nma[j]**2 + std_nma[i]**2)
        nma_5 = nma_d - 1.96 * nma_std
        nma_95 = nma_d + 1.96 * nma_std
        final_df.append([nodelabels[nodes[i]], nodelabels[nodes[j]], dir_d, dir_std, dir_5,
                         dir_95, ind_d, ind_std, ind_5, ind_95, ind_tau, ind_tausd,
                         nma_d, nma_std, nma_5, nma_95, tau_nma, tausd_nma])
        c += 1
        print()
    final_df = pd.DataFrame(final_df, columns=final_cols)
    if save_path is not None:
        final_df.to_csv(save_path)
    display(final_df.head(10))
    print(f"DIRECT-INDIRECT ESTIMATION DONE [tte = {(time.time() - start) // 60} mins]")


def dirIndZ(table_path):
    # this function performs the Z-test between direct and indirect estimates
    # adding the result as a column to the table generated by the function above
    # the table path itself is the input
    df = pd.read_csv(table_path)
    sigma = np.sqrt(df['direct_std'].values**2 + df['indirect_std'].values**2)
    z = (df['direct_est'].values - df['indirect_est'].values) / sigma
    df['Z_dir_ind'] = 2 * norm.cdf(-np.abs(z))
    df.to_csv(table_path, index=None)



# generates the funnel plots
def funnelPlots(pain=True, simple=True, ylims=(None, None), xlims=(None, None)):
    # if pain is true it imports the pain networks, else the walking-func ones
    # if simple it imports the table with no covariates, else with covariates
    # ylims and xlims delimit the axes in the plot
    studict = {'Beckwee 2015': 0.666, 'de Almeida 2019': 0.1, 'de Almeida 2020': 0.2,
           'Ettinger 1997': 0.3, 'Lim 2010': 0.4, 'Keogh 2018': 0.5, 'Mangione 1997': 0.6,
           'Moghadam 2017': 0.75, 'Oistad 2023': 0.8, 'Salacinski 2012': 0.9,
           'Samut 2015': 0.99}
    nodelabels = {'Education protocol': 'EP', 'Light-to-Moderate-to-Vigorous': 'LMV',
              'Light-to-Moderate': 'LM', 'Moderate': 'M', 'Moderate-to-Vigorous': 'MV',
              'No treatment': 'NT', 'Resistance exercise': 'RE', 'Vigorous': 'V',
              'Resistance exercise and Behavioral-lifestyle modification': 'REBLM',
              'Resistance, Aerobic, and Stretching exercises': 'REASE',
              '0_ No treatment': 'NT'}
    aerobics = ['LMV', 'LM', 'M', 'MV', 'V']
```

```
if pain:
    vartag = 'pain'
    indict = {0: 'Beckwee 2015', 1: 'de Almeida 2019', 2: 'Ettinger 1997', 3: 'Lim 2010',
              4: 'Oistad 2023', 5: 'Salacinski 2012', 6: 'Samut 2015'}
else:
    vartag = 'walk'
    indict = {0: 'de Almeida 2020', 1: 'Ettinger 1997', 2: 'Keogh 2018', 3: 'Mangione 1997',
              4: 'Moghadam 2017', 5: 'Salacinski 2012', 6: 'Samut 2015'}
if simple:
    baytag = 'simple'
else:
    baytag = 'covars'
raw_df = pd.read_csv(f"{out_path}{vartag}_edgelist.csv")
nma_df = pd.read_csv(f"{out_path}{vartag}_1v-{baytag}-gauss_resultable.csv")

figa, ax = plt.subplots(1, 1, figsize=(9, 6))
ax.yaxis.set_inverted(True)
olim = 2.5
ax.plot([0, olim], [0, 1.96 * olim], color='black', linestyle='--', alpha=0.5)
ax.plot([0, -olim], [0, 1.96 * olim], color='black', linestyle='--', alpha=0.5)
ax.plot([0, 0], [0, 1.96 * olim], color='black', linestyle='--', alpha=0.5)
for study, sdf in raw_df.groupby('study'):
    ax.scatter([], [], color=cm.gist_ncar_r(studict[indict[study]]), zorder=25,
               label=indict[study], edgecolor='black', linewidth=0.5)
    for _, line in sdf.iterrows():
        if line['T_a'] == '0_ No treatment':
            da = 0
            stda = 0
        else:
            da = nma_df[nma_df['variable'] == nodelabels[line['T_a']]]['mean'].values[0]
            stda = nma_df[nma_df['variable'] == nodelabels[line['T_a']]]['sd'].values[0]
        if line['T_b'] == '0_ No treatment':
            db = 0
            stdb = 0
        else:
            db = nma_df[nma_df['variable'] == nodelabels[line['T_b']]]['mean'].values[0]
            stdb = nma_df[nma_df['variable'] == nodelabels[line['T_b']]]['sd'].values[0]
        x = db - da - line['delta_ab']
        y = np.sqrt(line['std_ab']**2 + stda**2 + stdb**2)
        if nodelabels[line['T_a']] in aerobics and nodelabels[line['T_b']] in aerobics:
            symbol = '^'
        elif nodelabels[line['T_a']] not in aerobics and nodelabels[line['T_b']] not in aerobics:
            symbol = 'o'
        else:
            symbol = 's'
        ax.scatter(x, y, color=cm.gist_ncar_r(studict[indict[study]]), zorder=25,
                   marker=symbol, edgecolor='black', linewidth=0.5)
        if y < np.abs(x * 1.96):
            print(indict[study], nodelabels[line['T_a']], nodelabels[line['T_b']],
                  np.round(db - da, 3), np.round(line['delta_ab'], 3), np.round(y, 3))
ax.set_xlim(*xlims)
ax.set_ylim(*ylims)
ax.set_xlabel("Difference between network estimate and study-specific effect")
ax.set_ylabel("Error associated to the difference on the x-axis")
ax.legend(ncol=4, loc='upper center')
plt.savefig(f"{out_path}funnel-plots_{vartag}_{baytag}.pdf", bbox_inches='tight')
plt.show()
```
